# Supplementary material for: DRiPLOF: An RPL Extension for Multi-Interface Wireless Sensor Networks in Interference-Prone Environments
Source: Sensors (Basel). 2022 May 21;22(10):3906. doi: 10.3390/s22103906 (PMC9144939; doi:10.3390/s22103906)
Supplement: Supplementary file 1 [file sensors-22-03906-s001.zip › Supplementary/code/important.pdf]

Update Submodules

Because of size constraints for the supplementary files, we cloned the cooja and contiki-ng projects without updating the submodules. As such, prior to diving into the code, navigate to `Supplementary/code/contiki-ng/`, open a terminal (in this directory) and execute the following git command:

```
$ git submodule update --init --recursive
```

Now navigate to `Supplementary/code/cooja/` and execute the same git command. These commands may take a while to complete (especially in the case of contiki-ng).
