# Supplementary material for: DRiPLOF: An RPL Extension for Multi-Interface Wireless Sensor Networks in Interference-Prone Environments
Source: Sensors (Basel). 2022 May 21;22(10):3906. doi: 10.3390/s22103906 (PMC9144939; doi:10.3390/s22103906)
Supplement: Supplementary file 1 [file sensors-22-03906-s001.zip › Supplementary/code/instructions.pdf]

Dual-Radio Interface RPL (DRiPL)

This document describes the usage and configuration of the twofaced example for Contiki-NG. It is designed to work with Zolertia Zoul-based platforms and the Cooja simulator. The purpose of the twofaced application is to evaluate our implementation of a multi-interface RPL OF named DRiPLOF. If you make use of this example or any of its parts in your own research, we would like to ask you to include (in your own research output) a reference to the paper accompanying this example. The accompanying paper is:

"DRiPLOF: An RPL Extension for Multi-Interface Wireless Sensor Networks in Interference-Prone Environments" by R. Elsas, E. De Poorter, and J. Hoebeke (in submission)

Table of Contents

- Dual-Radio Interface RPL (DRiPL)
  - Table of Contents
  - Getting Started
  - Tips & Tricks
    - Custom Commands on Linux
  - Usage
    - Project Structure & Configuration
      - Physical Layer
      - Link Layer
      - Network Layer
      - Transport Layer
    - Simulation
      - Cooja
      - Experiments
      - Log Parsing & Visualization
  - Uncrustify
  - Recommended Resources
  - Contact and Affiliation
  - Licensing

Getting Started

The first step is to clone our remote repository to your local machine, updating all git submodules and checking out the `dripl` branch as follows:

```
$ cd ~
$ git clone https://github.com/tinstructor/contiki-ng.git
$ cd ~/contiki-ng/
$ git submodule update --init --recursive
$ git checkout dripl
```

If you'd like to adopt the proper git workflow (i.e., the forking workflow) for this project, according to the Atlassian git workflow tutorial, you'd first need to create your own remote repository (often named `upstream`) and local branch before pushing

that branch to your remote and setting the newly created local branch to track the corresponding branch of the remote. The following code snippet shows how this is done.

> ***Note:*** *there are shorter ways to achieve this, but the following snippet breaks it down into easy steps, sort off.*

```
$ cd ~/contiki-ng/
$ git remote add upstream <link to your remote>
$ git checkout -b <new branch>
$ git push upstream <new branch>
$ git branch -u upstream/<new branch>
```

The next step is to download the latest version of the ARM gcc compiler as follows:

```
$ cd ~
$ mkdir opt
$ cd opt/
$ wget https://developer.arm.com/-/media/Files/downloads/gnu-rm/10-2020q4/gcc-arm-none-eabi-10-2020-q4-major-x86_64-linux.tar.bz2
$ tar xjf gcc-arm-none-eabi-10-2020-q4-major-x86_64-linux.tar.bz2
$ sudo chmod -R -w gcc-arm-none-eabi-10-2020-q4-major
$ rm gcc-arm-none-eabi-10-2020-q4-major-x86_64-linux.tar.bz2
```

Now open up `.bashrc`:

```
$ cd ~
$ sudo nano .bashrc
```

The compiler must then be added to the `PATH` environment variable. Add the following line to end the of the file:

```
export PATH="$PATH:$HOME/opt/gcc-arm-none-eabi-10-2020-q4-major/bin"
```

However, before you can actually call the compiler you need to either restart your shel or execute the following command:

```
$ exec bash
```

You can check whether intallation was succesfull by requesting the version of the compiler as follows:

```
$ arm-none-eabi-gcc --version
arm-none-eabi-gcc (GNU Arm Embedded Toolchain 10-2020-q4-major) 10.2.1 20201103 (release)
Copyright (C) 2020 Free Software Foundation, Inc.
This is free software; see the source for copying conditions.  There is NO
warranty; not even for MERCHANTABILITY or FITNESS FOR A PARTICULAR PURPOSE.
```

The last few steps consist of installing some necessary packages:

```
$ sudo apt-get update
$ sudo apt-get install python3 python3-pip build-essential doxygen git curl wireshark python-serial srecord rlwrap pyserial uncrustify
```

When the Wireshark installer prompts you to say whether or not you want non-superusers to be able to capture packets select `"yes"` and finally add yourself to the `wireshark` group:

```
$ sudo usermod -a -G wireshark <user>
```

To be able to access the USB without using sudo, the user should be part of the groups plugdev and dialout.

```
$ sudo usermod -a -G plugdev <user>
$ sudo usermod -a -G dialout <user>
```

FINALLY you can start making changes to the code base. The twofaced application is located in `contiki-ng > examples > twofaced`. You might want to download VSCode as a convenient IDE for browsing through the code base. It comes with features that are very usefull when coding, especially in terms of code completion.

> ***Note:*** *for more information on the structure of the code base and how to modify it, have a look at the Contiki-NG Wiki.*

After a while, if you feel like the changes you've made are significant, you can always try and send us a merge request on GitHub (for the `dripl` branch). However, before you do that, make sure the changes you've made don't introduce conflicts. You may check this by pulling from the `dripl` branch on the `origin` remote and compiling + uploading. If the code doesn't compile or the flashed remote shows unexpected behavior, try and solve the underlying issue before sending a merge request. Also, it is important that you first uncrustify your code before submitting a merge request!

> ***Note:*** *a good entry book explaning git (and the tools it has to resolve conflicts) is Jump Start Git by Shaumik Daityari. More advanced topics are covered in the git bible, i.e., Pro Git which may be downloaded free of charge but can be hard to read for complete beginners.*

```
$ git pull origin dripl
$ cd ~/contiki-ng/examples/twofaced
$ make TARGET=zoul BOARD=firefly twofaced.upload
```

Alternatively, you may test the merged code (rebasing onto our dripl branch is also allowed and even preferred) by simulating the hardware in Renode. More about Renode in the corresponding section.

Tips & Tricks

I reckon you'll probably be working with Contiki-NG more often in the future. Hence you might as well make your life easier. For starters, since this application works with, e.g., the Zolertia Firefly, you should make use of the Makefile savetarget command, which allows you to save the target platform (and optionally a specific board revision) to a Makefile.target file as follows:

```
$ make TARGET=zoul BOARD=firefly savetarget
```

From now on, when you perform any make command from within the `contiki-ng > examples > twofaced` directory which requires to you specify the flash target, the configuration stored in the Makefile.target file is used if you don't explicitely specify a target.

```
$ make
using saved target 'zoul'
  MKDIR     build/zoul/firefly/obj
  CC        ../../arch/platform/zoul/./platform.c
  CC        ../../arch/platform/zoul/dev/leds-arch.c
  CC        ../../os/dev/leds.c
  ...
```

> ***Note:*** *Technically, the application probably works with other Zoul-based platforms with minimal changes to the code. The main difference will likely be in the pinout etc.*

Custom Commands on Linux

I'll do you a favor and briefly explain how to create Linux commands in order to make your life much easier. First, create a folder that you can use to store all self-defined shell scripts (because including custom commands in `/usr/bin/` is just asking for trouble):

```
$ cd ~
$ mkdir bin
```

Add the `~/bin/` folder to your `PATH` variable by appending the following line to `~/.bashrc`:

```
export PATH="$PATH:$HOME/bin"
```

Ok so now that's out of the way we'll create a command that lists all connected (via USB) boards or "motes" supported by Contiki-NG. More specifically, the command we'll create leverages some funtionality provided by the `~/contiki-ng/tools/motelist/motelist.py` script but makes it a bit more user-friendly. First, create a file in `~/bin/`:

```
$ cd ~/bin/
$ touch motelist
```

Next, add the following lines to `motelist`:

```
#!/bin/bash
python3 ~/contiki-ng/tools/motelist/motelist.py 2>&1
```

Then, we give the file all rights imaginable because we're only partially pedantic about good practices and sometimes (read: often) we only pretend to care:

```
$ cd ~/bin/
$ sudo chmod 777 motelist
```

Finally, restart your shell or execute the following convenient command so that the `PATH` variable is updated and now also points to `~/bin/`:

```
$ exec bash
```

Now, if you plugin (via USB) a board supported by Contiki-NG and run the `motelist` command, it'll list all sorts of useful information:

```
$ motelist
Port          Serial          VID     PID     Product                    Vendor
------------  --------------  ------  ------  -------------------------  ------------
/dev/ttyUSB0  ZOL-RM01-A0208  0x10C4  0xEA60  Zolertia RE-Mote platform  Silicon Labs
```

Usage

This example may be used to achieve several goals, each of which implies a slightly different way in which to use it. Nonetheless, in any case, compiling this example for a certain (supported) platform results in the creation of binaries for two different kinds of RPL nodes: non-root nodes and root nodes. When compiling for the Zolertia Firefly (as follows), the make command output lists all files that are output in this process:

```
$ cd ~/contiki-ng/examples/twofaced
$ make distclean
$ make TARGET=zoul BOARD=firefly
...
LD        build/zoul/firefly/twofaced-node.elf
OBJCOPY   build/zoul/firefly/twofaced-node.elf --> build/zoul/firefly/twofaced-node.i16hex
SREC_CAT  build/zoul/firefly/twofaced-node.i16hex --> build/zoul/firefly/twofaced-node.hex
OBJCOPY   build/zoul/firefly/twofaced-node.elf --> build/zoul/firefly/twofaced-node.bin
CP        build/zoul/firefly/twofaced-node.elf --> build/zoul/firefly/twofaced-node.zoul
CP        build/zoul/firefly/twofaced-node.zoul --> twofaced-node.zoul
CC        twofaced-root.c
LD        build/zoul/firefly/twofaced-root.elf
OBJCOPY   build/zoul/firefly/twofaced-root.elf --> build/zoul/firefly/twofaced-root.i16hex
SREC_CAT  build/zoul/firefly/twofaced-root.i16hex --> build/zoul/firefly/twofaced-root.hex
OBJCOPY   build/zoul/firefly/twofaced-root.elf --> build/zoul/firefly/twofaced-root.bin
CP        build/zoul/firefly/twofaced-root.elf --> build/zoul/firefly/twofaced-root.zoul
CP        build/zoul/firefly/twofaced-root.zoul --> twofaced-root.zoul
rm twofaced-node.o twofaced-root.o build/zoul/firefly/twofaced-root.i16hex build/zoul/firefly/obj/startup-gcc.o build/zoul/firefly/twofaced-node.i16hex
```

If, for some reason, you need direct access to the appropriate binaries for (in this case) the Zolertia Firefly (e.g., because you want to use them in conjunction with simulated hardware through Renode), you can thus find them in the `build/zoul/firefly/` directory. More specifically, `twofaced-node.elf` is the binary file for a non-root RPL node (as implemented in `twofaced-node.c`), while `twofaced-root.elf` is the binary file for a RPL root node (as implemented in `twofaced-root.c`). A similar output build directory is created if you compile for another (supported) platform.

> ***Note:*** *manually compiling is of little importance to you if you only wish to run a Cooja simulation, since the Cooja simulator GUI does this for you. Nonetheless, it*

> *is probably a good idea to have a basic understanding of how this works.*

Project Structure & Configuration

This project was written to enable trully multi-interfaced operation (specifically for RPL nodes but you could re-use the radio driver principles for other purposes just as well). In order for that to work, several additions / changes needed to be made to the Contiki-NG code base. Many of these changes closely align with the 3 bottom layers of the TCP / IP stack.

Physical Layer

Notice how `examples/twofaced/Makefile` contains the line `MODULES_REL += $(TARGET)/dev/twofaced-rf` . This means that when you execute the make command (for example) as `make TARGET=zoul BOARD=firefly`, the files contained in the directory `./zoul/dev/twofaced-rf` (notice this directory is relative w.r.t. the Makefile's directory) will be included during the build process. The purpose of this is to allow anybody to write a platform-specific radio driver for multi-interfaced operation.

> ***Note:*** *make sure `NETSTACK_CONF_RADIO` is set to `twofaced_rf_driver` in `examples/twofaced/project-conf.h`*

> ***Note:*** *when using Cooja, there are no underlying radio drivers. Instead, we modified `arch/platform/cooja/dev/cooja-radio.c` and `arch/platform/cooja/dev/cooja-radio.h` (i.e., the Cooja radio driver) to manipulate / read certain variables (largely) following the same principles as those laid out in this section. These variables are then accessed by the Cooja simulator through JNI. When compiling for the Cooja platfrom (which is automated by the Cooja GUI), there are no additional directories included during the build process because, again, there is no abstracting radio driver.*

A twofaced-rf radio driver module generally consists of a single source file (`twofaced-rf.c`), wherein the driver is actually implemented, and a single header file (`twofaced-rf.h`), mainly serving to declare radio driver functions such that they can be called from anywhere within the driver module's source file. Now, the purpose of a twofaced-rf driver is merely to serve as an abstraction on top of multiple existing radio drivers such that they may be used with minimal modifications. For example, each board in the Zoul platform family (such as the Firefly or RE-Mote) possesses both a cc2538 and a cc1200 radio. As such, since identifiers that are not names of variables or functions (e.g., structure tags) have no linkage by default, we declared all the possible underlying radio drivers in the beginning of `examples/twofaced/zoul/dev/twofaced-rf/twofaced-rf.c` with the storage class specifier `extern` (such that they have external linkage and hence represent the same objects as the drivers declared in `arch/cpu/cc2538/dev/cc2538-rf.h` and `arch/dev/radio/cc1200/cc1200.c`, which are also presented to the linker by virtue of them being declared with the storage class specifier `extern`).

```
/* The supported interface drivers */
extern const struct radio_driver cc2538_rf_driver;
extern const struct radio_driver cc1200_driver;
static const struct radio_driver *const available_interfaces[] =
TWOFACED_RF_AVAILABLE_IFS;
```

The drivers / interfaces that are actually used by the twofaced-rf abstraction driver are then configurable by means of a macro called `TWOFACED_RF_CONF_AVAILABLE_IFS` . If

you don't define it (for example in `examples/twofaced/project-conf.h`), the array of available interfaces will be initialized to `{ &cc2538_rf_driver, &cc1200_driver }`, meaning that both the cc2538 and cc1200 radio will be operational and the cc2538 radio (which is the first one in the list) shall start out as the preferred interface for all outgoing transmissions (more on that later).

> ***Note:*** *since `available_interfaces[]` is an array of const-qualified pointers to const-qualified `struct radio_driver` objects, nothing about the array nor its contents is modifiable after initialization (by means of list-initialization in this case)*

```
#ifdef TWOFACED_RF_CONF_AVAILABLE_IFS
#define TWOFACED_RF_AVAILABLE_IFS TWOFACED_RF_CONF_AVAILABLE_IFS
#else /* TWOFACED_RF_CONF_AVAILABLE_IFS */
#define TWOFACED_RF_AVAILABLE_IFS { &cc2538_rf_driver, &cc1200_driver }
#endif /* TWOFACED_RF_CONF_AVAILABLE_IFS */
```

The actual abstraction provided by a `twofaced_rf_driver` is predominantly centered auround setting the pointer `selected_interface` to the radio driver / interface to be used for outgoing transmissions, after which the driver passes most (not all) non-abstraction specific function calls to the selected underlying driver, takes the return value obtained from the underlying function call, and returns that same value. For example, the `transmit` function of the Zoul platform's `twofaced_rf_driver` looks like this:

```
static int
transmit(unsigned short transmit_len)
{
  return selected_interface->transmit(transmit_len);
}
```

Setting the selected interface of a twofaced-rf radio driver module can be achieved in two ways. Firstly, one could call the `twofaced_rf_driver`'s `set_value()` function and pass the `RADIO_PARAM_SEL_IF_ID` radio parameter with a value equal to the identifier (this is NOT the MAC address, which uniquely identifies the whole node and not its interfaces!) associated with the radio driver / interface you want to select. For example, `examples/twofaced/project-conf.h` sets the following interface identifiers (make sure they're unique and not 0):

```
#define CC2538_CONF_INTERFACE_ID 1
#define CC1200_CONF_INTERFACE_ID 2
```

Secondly, one could also call the `twofaced_rf_driver`'s `set_object()` function, while passing the `RADIO_PARAM_SEL_IF_DESC` radio parameter and a string literal equal to the `driver_descriptor` of a certain `struct radio_driver` object. However, since this parameter was newly added to `struct radio_driver` in `os/dev/radio.h`, it is not neccessarily initialized in every existing definition of a `const struct radio_driver` object (since this must be explicitly added to the initialization list). Note that this is also the case for several other newly added `struct radio_driver` parameters which are typically only used for twofaced-rf abstraction drivers. As such, the cc2538's radio driver now looks like this:

```
const struct radio_driver cc2538_rf_driver = {
  init,
  prepare,
  transmit,
  send,
  read,
  channel_clear,
  receiving_packet,
  pending_packet,
  on,
  off,
  get_value,
  set_value,
  get_object,
  set_object,
// The following are all newly added parameters:
  NULL,
  NULL,
  NULL,
  NULL,
  NULL,
  "cc2538_rf_driver"
};
```

> ***Note:*** *because of how list-initialization works, adding new parameters at the end of a struct declaration does not break existing functionality of other radio drivers of the type `struct radio_driver`. It does however mean that you need to put something (in our case all NULL pointers) at all positions in the initialization list prior to the last parameter you actually wish to initialize (or use member initialization instead).*

To make sure you can't change the selected interface in between two radio driver operations that MUST be performed on the same underlying radio driver (such as, e.g., consecutive calls to `prepare()` and `transmit()`), we've introduced a mutex lock (have a look at `mutex_try_lock()` and `mutex_unlock()` in `os/sys/mutex.h`) that may be (try-) locked and unlocked by means of calling the `twofaced_rf_driver`'s `lock_interface()` and `unlock_interface()` functions respectively whenever you want to prevent the selected interface from changing in a certain region.

```
const struct radio_driver twofaced_rf_driver = {
  init,
  prepare,
  transmit,
  send,
  read,
  channel_clear,
  receiving_packet,
  pending_packet,
  on,
  off,
  get_value,
  set_value,
```

```
  get_object,
  set_object,
  lock_interface,
  unlock_interface,
  channel_clear_all,
  receiving_packet_all,
  pending_packet_all,
  "twofaced_rf_driver"
};
```

> *More information available upon request.*

Link Layer

Contrary to the generally accepted process for defining a new MAC layer in Contiki-NG (as defined here), we do not set the `MAKE_MAC` make variable to `MAKE_MAC_OTHER` in `examples/twofaced/Makefile`. Instead, we do the following:

```
MAKE_MAC = MAKE_MAC_TWOFACED

ifeq ($(MAKE_MAC),MAKE_MAC_TWOFACED)
  MODULES_REL += net/mac/twofaced-mac
  CFLAGS += -DMAC_CONF_WITH_TWOFACED=1
endif
```

However, we still put the following in `examples/twofaced/project-conf.h`:

```
#define NETSTACK_CONF_MAC twofaced_mac_driver
```

This allows us to perform preprocessor checks on `MAC_CONF_WITH_TWOFACED` rather than `MAC_CONF_WITH_OTHER` and includes the module required to run the twofaced mac protocol only when necessary. This MAC protocol is an adaptation of the CSMA MAC protocol with some multi-interface specific changes / additions. Note that when `MAKE_MAC` is set to `MAKE_MAC_TWOFACED` (in `examples/twofaced/Makefile`), the contents of the `examples/twofaced/net/mac/twofaced-mac` directory are included in the build path. The files in this directory make up the Twofaced MAC protocol (the driver is in there somewhere). In addition, a `CFLAG` is added. This ensures that the macro `MAC_CONF_WITH_TWOFACED` is defined and set to `1` accross the whole of Contiki-NG (which is pretty usefull for performing some checks) prior to any other macros being defined (at least those that aren't passed as `CFLAG`). As such, `examples/twofaced/project-conf.h` allows us to automatically set the MAC driver to `twofaced_mac_driver` when setting `MAKE_MAC` to `MAKE_MAC_TWOFACED`, because doing so includes the correct driver files and defines `MAC_CONF_WITH_TWOFACED` (this is pretty nifty stuff innit):

```
#if MAC_CONF_WITH_TWOFACED
...
#define NETSTACK_CONF_MAC twofaced_mac_driver
...
#endif /* MAC_CONF_WITH_TWOFACED */
```

The MAC driver interface for the Twofaced MAC (i.e., `twofaced_mac_driver`, which points to a standardized set of functions) is defined in `examples/twofaced/net/mac/twofaced-mac/twofaced-mac.c`. The general idea is very

similar to the PHY driver modifications in that all MAC drivers are of the type `struct mac_driver`, which allows us to capitalize on list initialization. More specifically, we can safely add new parameters at the end of a struct declaration without breaking existing functionality of other radio drivers of the same type (i.e., `struct mac_driver` in this case). As such, we've added 2 new function pointers to `lock_input()` and `unlock_input()` respectively. When using the Twofaced MAC, these functions must be used by the actual radio drivers (not the abstraction radio driver) to ensure every call to the MAC driver's `input()` function completes and returns prior to it being called again by another interface's radio driver.

> ***Note:*** *the functions pointed to by the MAC driver can be accessed through the* `NETSTACK_MAC` *macro. For example, the selected MAC protocol is initialized in* `os/net/netstack.c` *by calling* `NETSTACK_MAC.init()`*.*

```
const struct mac_driver twofaced_mac_driver = {
  "twofaced_mac",
  init,
  send,
  input,
  on,
  off,
  max_payload,
  lock_input,
  unlock_input,
};
```

For example, in the `pollhandler()` of `arch/dev/radio/cc1200/cc1200.c`, it is clear that when a packet is available, the input lock of the MAC is first tried (if the Twofaced MAC is enabled, which signifies multi-interface operation) before writing the contents of the packet to the **common** (i.e., to all interfaces) packet buffer and calling the MAC's `input()` function. The same actions are (more or less) performed in `arch/cpu/cc2538/dev/cc2538-rf.c` and `arch/platform/cooja/dev/cooja-radio.c` (although that last one is slightly different).

> ***Note:*** *I know performing a preprocessor check on* `MAC_CONF_WITH_TWOFACED` *as the only way to check for multi-interface operation is probably a little janky but I did it anyway.*

```
static void
pollhandler(void)
{
  //...
  if((rx_mode_value & RADIO_RX_MODE_POLL_MODE) == 0 && rx_pkt_len > 0) {
    int len;

#if MAC_CONF_WITH_TWOFACED
    if(NETSTACK_MAC.lock_input()) {
      /* MAC input lock acquired by cc1200 */
#endif /* MAC_CONF_WITH_TWOFACED */
    packetbuf_clear();
    len = read(packetbuf_dataptr(), PACKETBUF_SIZE);

    if(len > 0) {
```

```
      packetbuf_set_datalen(len);
      NETSTACK_MAC.input();
    }
#if MAC_CONF_WITH_TWOFACED
      NETSTACK_MAC.unlock_input();
      /* MAC input lock released by cc1200 */
    } else {
      /* Failed trying MAC input lock, polling process again */
      process_poll(&cc1200_process);
    }
#endif /* MAC_CONF_WITH_TWOFACED */
  }
}
```

> *More information available upon request.*

This brings us to the way in which Contiki-NG keeps statistics about links to neighbors. More specifically, it keeps track of per-neighbor link-statistics by storing value entries of type `struct link_stats` in a Contiki-NG `NBR_TABLE` called `link_stats` (see `os/net/link-stats.c`). Upon successfully transmitting a (unicast) packet, the function `link_stats_packet_sent()` is called (defined in `os/net/link-stats.c` and declared in `os/net/link-stats.h`). Here, the link_stats table is checked for an existing entry keyed on the link-layer address of the neighbor to which the packet was sent. If no existing entry is found, a new entry in the link_stats neighbor table is added. If adding the new entry was succesful, a linked-list (consisting of structs of a certain format, see `os/lib/list.h`) named `interface_list`, which is a member of a `link_stats` struct object (and thus of each link-stats neighbor table entry), can be initialized. Next, whether or not the entry was newly added doesn't really matter, the `interface_list` of the given link-stats table entry (i.e., the one we found or otherwise newly added just now) is checked for an entry (of the type `struct interface_list_entry`) containing the identifier (this is NOT a MAC address) of the interface that was put in the packetbuffer attribute `PACKETBUF_ATTR_INTERFACE_ID` by a higher stack layer before transmitting (see [this wiki article]() for more information on Contiki-NG buffers and how to use them). If such entry was found (i.e., `interface_list_entry_from_id()` didn't return `NULL`), the existing interface list entry is updated. Otherwise, when no interface list entry is found for the given interface identifier, a new entry is added if and only if the current number of entries in the link-stats table entry's interface list is less than `LINK_STATS_NUM_INTERFACES_PER_NEIGHBOR`, i.e., the amount of interfaces each node in the network is expected to have (this constant is also used for normalized metric calculation etcetera).

> ***Note:*** *a Contiki-NG `NBR_TABLE` is pretty straightforward once you get the hang of it. Have a look at `os/net/nbr-table.h`. If you don't speak fluent preprocessor hocus-pocus, don't worry. What it boils down to is that each entry in a neighbor table is keyed on the link-layer address of a neighbor and stores entries of a given type.*

> ***Note:*** *The defer flag system is part of POOF operation and it is effectively ignored when setting the `LINK_STATS_CONF_PREF_IF_METRIC_ONLY` macro (to 1) in `examples/twofaced/project-conf.h`, which is absolutely paramount when using DRiPLOF!!! Note that the defer flag in an interface table entry must not be mistaken for an accurate representation of whether or not a physical link is down!*

> *Its sole purpose is to facilitate the stability mechanism of POOF. Anyhow, the POOF rules for the defer flag (which is a system we developed to accomodate for POOF, as this information was severely lacking in the paper we derived it from) are as follows: 1) The defer flag is always reset after path cost computation (during an OF call), regardless of the stored inferred metric in the interface table entry; 2) The link-layer may only reset the defer flag when the inferred metric updates and is no longer worse than the metric threshold; 3) The link-layer may only set the defer flag when the inferred metric updates and becomes worse than the metric threshold; and 4) When an inferred metric is not updated, or when it is but it doesn't cross the metric threshold in any direction, the link-layer may not update the corresponding defer flag.*
>
> *As such, when during path cost computation for the preferred parent (and only for the preferred parent!), POOF sees one out of two defer flags is set (or any number out of `LINK_STATS_NUM_INTERFACES` except for 0 and `LINK_STATS_NUM_INTERFACES`), path cost for the preferred parent is computed based on the inferred metric calculated during the previous OF call. Again, not that this only relevant to our implementation of POOF.* ***WHEN USING DRIPLOF, YOU MUST SET `LINK_STATS_CONF_PREF_IF_METRIC_ONLY` TO 1*** *in `examples/twofaced/project-conf.h`, which effectively disables defer flag checking (and enables some other things too, more on that later).*

```
void
link_stats_packet_sent(const linkaddr_t *lladdr, int status, int numtx)
{
  struct interface_list_entry *ile;
  struct link_stats *stats;
  ...
  stats = nbr_table_get_from_lladdr(link_stats, lladdr);
  if(stats == NULL) {
    /* If transmission failed, do not add the neighbor, as the neighbor might not
exist anymore */
    if(status != MAC_TX_OK) {
      return;
    }

    /* Add the neighbor */
    stats = nbr_table_add_lladdr(link_stats, lladdr, NBR_TABLE_REASON_LINK_STATS,
NULL);
    if(stats != NULL) {
      /* Init node-scope metric (irrelevant for twofaced) */
      ...
    } else {
      return; /* No space left, return */
    }
    LIST_STRUCT_INIT(stats, interface_list);
  }

  uint8_t if_id = packetbuf_attr(PACKETBUF_ATTR_INTERFACE_ID);
  ile = interface_list_entry_from_id(stats, if_id);
  if(ile != NULL) {
    /* Update the existing ile */
    uint16_t old_metric = ile->inferred_metric;
```

```
    /*  */
    ile->inferred_metric = LINK_STATS_INFERRED_METRIC_FUNC(ile, status, numtx,
LINK_STATS_METRIC_INIT_FLAG_FALSE);
    ...
    /* When an inferred metric is not updated, or when it is but it doesn't
       cross the metric threshold in any direction, the link-layer may not
       update the corresponding defer flag */
    if(old_metric != ile->inferred_metric) {
      if(LINK_STATS_WORSE_THAN_THRESH(old_metric) &&
        !LINK_STATS_WORSE_THAN_THRESH(ile->inferred_metric)) {
        ile->defer_flag = LINK_STATS_DEFER_FLAG_FALSE;
        ...
      } else if(!LINK_STATS_WORSE_THAN_THRESH(old_metric) &&
                LINK_STATS_WORSE_THAN_THRESH(ile->inferred_metric)) {
        ile->defer_flag = LINK_STATS_DEFER_FLAG_TRUE;
        ...
      }
      /* It makes no sense to re-select the preferred interface if there's
         no change in inferred metric for the given interface (represented
         by the ile) */
      link_stats_select_pref_interface(lladdr);
    }
  } else {
    if(list_length(stats->interface_list) < LINK_STATS_NUM_INTERFACES_PER_NEIGHBOR) {
      /* Create new ile and add to interface list */
      ile = memb_alloc(&interface_memb);
      if(ile != NULL) {
        ile->if_id = if_id;
        /*  */
        ile->inferred_metric = LINK_STATS_INFERRED_METRIC_FUNC(ile, status, numtx,
LINK_STATS_METRIC_INIT_FLAG_TRUE);
        list_add(stats->interface_list, ile);
        ...
        link_stats_select_pref_interface(lladdr);
      } else {
        ...
        return;
      }
    }
  }
  ...
  /* Update last timestamp and freshness */
  ...
  /* Other stuff */
  ...
}
```

> ***Note:*** *a similar function named `link_stats_input_callback()` is called when a packet is received from a neighbor. Since the functionality provided by this function with regard to multi-interfaced operation is almost (not completely) identical to `link_stats_packet_sent()` you should be able to understand how it works by just looking at `os/net/link-stats.c`. Hence, we shall not discuss it any further.*

Notice how the inferred metric stored in an ile is set to `LINK_STATS_INFERRED_METRIC_FUNC()`? This allows you to configure the function used to obtain and / or calculate said inferred metric by means of the macro `LINK_STATS_CONF_INFERRED_METRIC_FUNC` as can be seen in `os/net/link-stats.h`:

```
/* Function used to retrieve inferred metric */
#ifdef LINK_STATS_CONF_INFERRED_METRIC_FUNC
#define LINK_STATS_INFERRED_METRIC_FUNC LINK_STATS_CONF_INFERRED_METRIC_FUNC
#else /* LINK_STATS_CONF_INFERRED_METRIC_FUNC */
#if LINK_STATS_INTERFACES_WITH_ETX
#define LINK_STATS_INFERRED_METRIC_FUNC( ile, status, numtx, mi_flag )
get_interface_etx(ile, status, numtx, mi_flag)
#else /* LINK_STATS_INTERFACES_WITH_ETX */
#define LINK_STATS_INFERRED_METRIC_FUNC( ile, status, numtx, mi_flag )
guess_interface_lql_from_rssi(ile, status)
#endif /* LINK_STATS_INTERFACES_WITH_ETX */
#endif /* LINK_STATS_INFERRED_METRIC_FUNC */
```

In case of the default configuration, the inferred metric uses a very basic function to calculate the LQL from the RSSI value currently contained in the `packetbuf` (and thus you should make sure to only call this function when there is an actual packet in `packetbuf`). That is, if the transmit status passed to the function (which is named `guess_lql_from_rssi()` by the way) equals `MAC_TX_OK`. Otherwise, it shall return an LQL value of 0, indicating the LQL is unavailable and the link should be considered down.

> ***Note:*** *in practice, you probably want to set the macro `LINK_STATS_INTERFACES_WITH_ETX` in `examples/twofaced/project-conf.h`, as we did most of the time. Since LQL is not really additive, you shouldn't really use it for DRiPLOF anyway. The function `get_interface_etx()` is quite a bit more complicated than `guess_interface_lql_from_rssi()`, but you should be able to understand it. After all, it operates nearly identically to how Contiki-NG performs ETX calculations for neighbors rather than interfaces (which is our addition).*

```
uint16_t
guess_interface_lql_from_rssi(const struct interface_list_entry *ile, int status)
{
  if(ile != NULL && status == MAC_TX_OK) {
    uint16_t lql;
    int16_t bounded_rssi = (ile->rssi == 0) ? packetbuf_attr(PACKETBUF_ATTR_RSSI) :
ile->rssi;
    bounded_rssi = MIN(bounded_rssi, RSSI_HIGH);
    bounded_rssi = MAX(bounded_rssi, RSSI_LOW + 1);
    lql = 7 - ((((bounded_rssi - RSSI_LOW) * 6) + RSSI_DIFF / 2) / RSSI_DIFF);
    ...
    return lql;
  }
  return 0;
}
```

The inferred metric stored in the entries of link-stats table entry's interface list is primarily used for two purposes, i.e., selecting the preferred interface for communication with a given neighbor (i.e., the neighbor represented by a link-stats

table entry) and calculating the normalized metric towards that same neighbor. Preferred interface selection is handled by the function `link_stats_select_pref_interface()` in `os/net/link-stats.c`.

More specifically, it looks for an existing entry in the `link_stats` neighbor table by means of the supplied link-layer address. If such entry is found, it iterates through said entry's interface list (which is a linked list, see `os/lib/list.h` for more info) and compares each ile's inferred metric with the inferred metric stored in the ile that currently represents the preferred interface towards the neighbor represented by the link-stats table entry containing the interface list of which it is part. Then, if both interfaces are down (as indicated by `LINK_STATS_WORSE_THAN_THRESH`) or if both interfaces are up, and `LINK_STATS_WORSE_THAN_THRESH` is not defined as `< LINK_STATS_METRIC_THRESHOLD`, metric comparison is based on the actual value of the `inferred_metric` parameter stored in each ile. However, if both interfaces are down and `LINK_STATS_WORSE_THAN_THRESH` is indeed defined as `< LINK_STATS_METRIC_THRESHOLD`, then comparison is still performed but the outcome will always be the same (i.e., the first interface in the neighbor's interface list will remain / become preferred). The latter is a remnant of an old implementation and will probably be simplified in future releases.

At the end of each iteration, if `if_metric < pref_if_metric`, the `pref_ile` is set to `ile`.

```
int
link_stats_select_pref_interface(const linkaddr_t *lladdr)
{
  struct link_stats *stats;
  stats = nbr_table_get_from_lladdr(link_stats, lladdr);
  if(stats == NULL) {
    ...
    return 0;
  }
  struct interface_list_entry *ile, *pref_ile;
  pref_ile = list_head(stats->interface_list);
  ile = list_item_next(pref_ile);
  while(ile != NULL) {
    uint32_t pref_if_metric;
    uint32_t if_metric;
    if(LINK_STATS_WORSE_THAN_THRESH(ile->inferred_metric) ==
LINK_STATS_WORSE_THAN_THRESH(pref_ile->inferred_metric)) {
      if(LINK_STATS_WORSE_THAN_THRESH(ile->inferred_metric)) {
        /* Both interfaces are down */
        if(LINK_STATS_WORSE_THAN_THRESH(LINK_STATS_METRIC_THRESHOLD - 1)) {
          /* If worse than threshold is defined as "< threshold", use placeholder
values */
          pref_if_metric = LINK_STATS_METRIC_PLACEHOLDER;
          if_metric = LINK_STATS_METRIC_PLACEHOLDER;
        } else {
          /* If worse than threshold is defines as "> threshold", use actual values */
          pref_if_metric = pref_ile->inferred_metric;
          if_metric = ile->inferred_metric;
        }
      } else {
```

```
        /* Both interfaces are up, use actual values */
        pref_if_metric = pref_ile->inferred_metric;
        if_metric = ile->inferred_metric;
      }
      /* If metric of next interface is better than metric of pref if, new pref if */
      pref_ile = (if_metric < pref_if_metric) ? ile : pref_ile;
    } else if(LINK_STATS_WORSE_THAN_THRESH(pref_ile->inferred_metric)) {
      /* The next if is better simply because it is up and the currently pref if is
down! */
      pref_ile = ile;
    }
    ile = list_item_next(ile);
  }
  if(stats->pref_if_id != pref_ile->if_id) {
    ...
    stats->pref_if_id = pref_ile->if_id;
  }
  return 1;
}
```

As said before, the inferred metric stored in the entries of link-stats table entry's interface list is primarily used for two purposes: 1) preferred interface selection; and 2) metric normalization. When the normalized metric of a neighor is updated by calling `link_stats_update_norm_metric()` in `os/net/link-stats.c` (an action that is exclusively coordinated by the routing layer), what actually happens next depends on the value of `LINK_STATS_PREF_IF_METRIC_ONLY`, which indicates that defer flag mechanism should be disabled, and that we base the normalized metric of a neighbor on the inferred metric of the preferred interface towards said neighbor only (as opposed to basing it on the inferred metric of all interfaces towards said neighbor). Which approach is right depends on the routing layer configuration (more on that later).

```
int
link_stats_update_norm_metric(const linkaddr_t *lladdr)
{
  struct link_stats *stats;
  stats = nbr_table_get_from_lladdr(link_stats, lladdr);
  if(stats == NULL) {
    ...
    return 0;
  }
  struct interface_list_entry *ile;
  uint8_t num_if = 0;
  uint8_t num_worse = 0;
  uint16_t old_norm = stats->normalized_metric;
#if LINK_STATS_PREF_IF_METRIC_ONLY
  ile = list_head(stats->interface_list);
  while(ile != NULL) {
    if(LINK_STATS_WORSE_THAN_THRESH(ile->inferred_metric)) {
      num_worse++;
    }
    num_if++;
    ile = list_item_next(ile);
```

```
  }
  if(num_if > LINK_STATS_NUM_INTERFACES_PER_NEIGHBOR) {
    goto illegal_num_if;
  }
  if(num_worse >= LINK_STATS_NUM_INTERFACES_PER_NEIGHBOR) {
    goto all_if_down;
  }
  ile = interface_list_entry_from_id(stats, stats->pref_if_id);
  if(ile != NULL) {
    stats->normalized_metric = LINK_STATS_NORM_METRIC_FUNC(ile->inferred_metric,
                                                           num_if - num_worse);
  } else {
    ...
    stats->normalized_metric = 0xffff;
  }
#else /* LINK_STATS_PREF_IF_METRIC_ONLY */
  ile = list_head(stats->interface_list);
  uint64_t numerator = 0;
  uint32_t denominator = 0;
  while(ile != NULL) {
    uint32_t inferred_metric;
    if(LINK_STATS_WORSE_THAN_THRESH(ile->inferred_metric)) {
      inferred_metric = LINK_STATS_METRIC_PLACEHOLDER;
      num_worse++;
    } else {
      inferred_metric = ile->inferred_metric;
    }
    numerator += inferred_metric;
    denominator++;
    num_if++;
    ile = list_item_next(ile);
  }
  if(num_if > LINK_STATS_NUM_INTERFACES_PER_NEIGHBOR) {
    goto illegal_num_if;
  }
  if(num_worse >= LINK_STATS_NUM_INTERFACES_PER_NEIGHBOR) {
    goto all_if_down;
  }
  uint8_t num_if_left = LINK_STATS_NUM_INTERFACES_PER_NEIGHBOR - num_if;
  if(num_if_left > 0) {
    numerator += (num_if_left * LINK_STATS_METRIC_PLACEHOLDER);
    denominator += num_if_left;
  }
  /* Never divide by zero or the universe might implode */
  denominator = denominator ? denominator : 1;
  /* Integer division but rounded to nearest integer */
  stats->normalized_metric = MIN((numerator + denominator / 2) / denominator,
UINT16_MAX);
#endif /* LINK_STATS_PREF_IF_METRIC_ONLY */
  ...
  return 1;
```

```
all_if_down:
  ...
  stats->normalized_metric = 0xffff;
  return 0;

illegal_num_if:
  ...
  stats->normalized_metric = 0xffff;
  return 0;
}
```

Now, if `LINK_STATS_PREF_IF_METRIC_ONLY` is set, metric normalization is performed after some checks that simply make sure the number of interfaces, and the number of those for which the inferred metric is worse than threshold, makes sense. Note that, at this stage, if all interfaces of a neighbor are found to be unavailable (i.e., they have worse-than-threshold inferred metrics), the neighbor is considered unavailable in its entirety and its normalized metric automatically defaults to `0xffff`. The actual function used for metric normalization is configurable by setting `LINK_STATS_CONF_NORM_METRIC_FUNC` to a different function (of your own design). Note that this only applies to the case when `LINK_STATS_PREF_IF_METRIC_ONLY` is set!

```
#ifdef LINK_STATS_CONF_NORM_METRIC_FUNC
#if LINK_STATS_PREF_IF_METRIC_ONLY
#define LINK_STATS_NORM_METRIC_FUNC LINK_STATS_CONF_NORM_METRIC_FUNC
#else /* LINK_STATS_PREF_IF_METRIC_ONLY */
#error "Defining a normalized metric function is only supported when
LINK_STATS_PREF_IF_METRIC_ONLY == 1"
#endif /* LINK_STATS_PREF_IF_METRIC_ONLY */
#else /* LINK_STATS_CONF_NORM_METRIC_FUNC */
#if LINK_STATS_PREF_IF_METRIC_ONLY
#define LINK_STATS_NORM_METRIC_FUNC( preferred_metric, valid_links )
get_valid_link_norm(preferred_metric, valid_links)
#endif /* LINK_STATS_PREF_IF_METRIC_ONLY */
#endif /* LINK_STATS_NORM_METRIC_FUNC */
```

By default (that is, when `LINK_STATS_PREF_IF_METRIC_ONLY == 1`), `get_valid_link_norm()` is used for calculating the normalized metric of a neighbor. It accepts, as a function parameter, the inferred metric of the preferred interface towards a given neighbor, together with the number of available interfaces towards that same neighbor:

```
uint16_t
get_valid_link_norm(uint16_t preferred_metric, uint8_t valid_links)
{
  if(LINK_STATS_WORSE_THAN_THRESH(preferred_metric) ||
     valid_links > LINK_STATS_NUM_INTERFACES_PER_NEIGHBOR ||
     valid_links == 0) {
    return 0xffff;
  }

  uint8_t invalid_links = LINK_STATS_NUM_INTERFACES_PER_NEIGHBOR - valid_links;
  if(invalid_links > LINK_STATS_MAX_INVALID_LINKS) {
    return 0xffff;
  }
```

```
  double weight = MIN(invalid_links, LINK_STATS_MAX_INVALID_LINKS) /
(double)LINK_STATS_INVALID_LINKS_DIV;
  return weight * LINK_STATS_SCALE_MULTIPLIER + preferred_metric * (1.0 - weight) +
0.5;
}
```

Aforementioned function is part of the requirements laid out in our DRiPLOF specification, which is an extension to the RPL routing layer (POOF is a similar extension designed by different people but implemented by us) (more on that later). Note that, in Contiki-NG, metric normalization is abstracted from the routing layer.

Network Layer

While technically redundant for the `MAKE_NET` variable (because it is configured like that by default in `Makefile.include`), we set the following make variables in `examples/twofaced/Makefile` (partially for the sake of clarity):

```
MAKE_NET = MAKE_NET_IPV6
MAKE_ROUTING = MAKE_ROUTING_RPL_CLASSIC
```

The choice for RPL-classic over RPL-lite has a lot to do with the feature-completeness of RPL-classic (which, frankly, is abominable, but RPL-lite is even worse ***deep sigh to signify I'm deeply disappointed***) and the fact that it supports all MOPs (instead of just non-storing mode). As you undoubtedly know, much of the functionality provided by RPL is governed by the objective function. Hence, a large part of our efforts towards truly multi-interfaced RPL nodes consisted of developing new objective functions. Much like with our multi-interface radio driver abstraction (well let's say we borrowed those principles from here) `os/net/routing/rpl-classic/rpl-dag.c` declares all possible objective functions a RPL node could run with the storage class specifier `extern`:

> ***Note:*** *a RPL objective function that is declared without a storage class specifier automatically has external linkage because it is of type* `rpl_of_t` *and not* `struct rpl_of`*. Hence,* `os/net/routing/rpl-classic/rpl-of0.c` *for example, simply declares (and simultaneously defines) OF0 as* `rpl_of_t rpl_of0 = {<init list>}`*. However, in* `os/net/routing/rpl-classic/rpl-dag.c` *we must still use the* `extern` *keyword for reasons that currently escape me.*

```
extern rpl_of_t rpl_of0, rpl_mrhof, rpl_poof, rpl_driplof;
static rpl_of_t * const objective_functions[] = RPL_SUPPORTED_OFS;
```

The objective functions that may actually be used by a RPL node are then configurable by means of a macro called `RPL_CONF_SUPPORTED_OFS` (see `os/net/routing/rpl-classic/rpl-conf.h`). Put differently, the `RPL_CONF_SUPPORTED_OFS` macro (which should be defined as an array initialization list) configures the OFs supported by any node (root or non-root) at runtime. A node may only join a RPL instance (advertised in a DIO) which is based on one of the OFs in this list as indicated by the OCP in the DODAG Configuration option attached to a DIO. If you don't define it (for example in `examples/twofaced/project-conf.h`), the array of supported objective functions will be initialized to `{ &rpl_mrhof }`, meaning that a node will only understand MRHOF.

```
#ifdef RPL_CONF_SUPPORTED_OFS
#define RPL_SUPPORTED_OFS RPL_CONF_SUPPORTED_OFS
```

```
#else /* RPL_CONF_SUPPORTED_OFS */
#define RPL_SUPPORTED_OFS {&rpl_mrhof}
#endif /* RPL_CONF_SUPPORTED_OFS */
```

Similarly, the OF that is disseminated through the network by root nodes (i.e., through the OCP field of DODAG Configuration options attached to DIOs) is configurable by means of a macro called `RPL_CONF_OF_OCP` (see `os/net/routing/rpl-classic/rpl-conf.h`). If you don't define it (for example, in `examples/twofaced/project-conf.h`), a node configured as root will set the OCP of DODAG Configuration options to `RPL_OCP_MRHOF` (i.e., 0x01). The OCP setting has no meaning to non-root nodes, as they'll run any OF advertised by the root as long as they recognize the corresponding OCP value and thus support a given OF.

```
#ifdef RPL_CONF_OF_OCP
#define RPL_OF_OCP RPL_CONF_OF_OCP
#else /* RPL_CONF_OF_OCP */
#define RPL_OF_OCP RPL_OCP_MRHOF
#endif /* RPL_CONF_OF_OCP */
```

OF drivers are to be defined in separate source files, for example `os/net/routing/rpl-classic/rpl-driplof.c` implements our multi-interface DRiPLOF driver and intializes a struct of type `rpl_of_t` (which has external linkage) with a standard set of function pointers to be used by RPL. One of these functions, `parent_link_metric(rpl_parent_t *p)`, retrieves a metric towards the supplied parent. For single-interface OFs, such as MRHOF (see `os/net/routing/rpl-classic/rpl-mrhof.c`) and OF0 (see `os/net/routing/rpl-classic/rpl-of0.c`), the metric retrieved is `stats->etx`, i.e., the overall ETX towards the neighbor. For multi-interfaced OFs, the metric retrieved is `stats->normalized_metric`, i.e., the result of the last call to `link_stats_update_norm_metric()`.

> ***Note:*** *the reason for the different metric retrieved by single- and multi-interfaced OFs is that doing it this way allowed us to keep existing functionality in the link stats table. However, one might as well let single-interface OFs also retrieve* `stats->normalized_metric` *and remove all old link-stats functionality altogether. Some minimal tweaking is probably required though. After all, all legacy link stats functionality may be replicated with the multi-interface link stats functionality simply by setting some macros (such that the outcome would be identical).*

```
static uint16_t
parent_link_metric(rpl_parent_t *p)
{
  if(p == NULL) {
    return 0xffff;
  }

  const struct link_stats *stats = rpl_get_parent_link_stats(p);
  return stats != NULL ? stats->normalized_metric : 0xffff;
}
```

As mentioned before `link_stats_update_norm_metric()` (see `os/net/link-stats.c`) is exclusively called by the routing layer.

> *More information available upon request.*

I would like to state that Contiki-NG often takes liberties and that the liberties taken are generally useful. **Not so with the routing layers!** More specifically, there was one "simplification" made to the RPL-Classic implementation wherein nodes always advertised the rank computed for the path through its preferred parent, regardless of the governing OF. While this is correct behavior for OF0 (and derivatives), this shows a complete disregard for at least half the potential of MRHOF (and derivatives). More specifically, for MRHOF and its derivatives, the rank to be advertised must be set to the max of 3 following values: 1) The rank computed for the path through the preferred parent; 2) The highest rank advertised by any of its parent set members (this is NOT the same as the computed rank for the path through said node), rounded to the next higher integral rank; and 3) The largest computed rank among paths through the parent set, minus MaxRankIncrease. Many people think that MRHOF is all about the preferred parent hysteresis but I would argue that it's at least as much about aforemention point 2, because it prevents local repairs (which are incredibly costly). More specifically, it makes sure that advertised ranks are reasonably conservative, allowing nodes to keep larger parent sets. Long story short: I attempted to fix this major fault.

> ***Note:*** *I am sure that my attempted fix is an equally hot mess to the prior implementation but now it is at least closer to performing as intended.*

Let's have a look at the new OF driver for MRHOF. Notice the addition of the function pointers to `parent_is_acceptable()` (more about that later) and `rank_via_dag()`.

> ***Note:*** *all OF drivers set these function pointers and implement the corresponding functions.*

```
rpl_of_t rpl_mrhof = {
  reset,
#if RPL_WITH_DAO_ACK
  dao_ack_callback,
#endif
  parent_link_metric,
  parent_has_usable_link,
  parent_is_acceptable,
  parent_path_cost,
  rank_via_parent,
  best_parent,
  best_dag,
  update_metric_container,
  rank_via_dag,
  RPL_OCP_MRHOF
};
```

The function `rank_via_dag(rpl_dag_t *dag, linkaddr_t *blame)` effectively implements the 3 point advertised rank calculation. Moreover, it allows the function caller to know which parent is to blame for the rank value returned by `rank_via_dag()` (or `rpl_rank_via_dag()`, but that's just a handy wrapper, see `os/net/routing/rpl-classic/rpl-dag.c`).

> *More information available upon request.*

Transport Layer

When the execution flow (when transmitting a packet) inevitably ends up in `tcpip_ipv6_output()` (see `os/net/ipv6/tcpip.c`) and encounters an unconditional jump in the form of a `goto send_packet;` statement, the `uip_buf` flags are checked to see if the `UIPBUF_ATTR_FLAGS_ALL_INTERFACES` flag is set (see `os/net/routing/rpl-classic/rpl-icmp6.c` and `os/net/routing/rpl-classic/rpl-timers.c` for examples of when that happens). If it is not set, meaning the packet to be transmitted must be sent to the given `nbr` via the preferred interface for that neighbor, the link-stats table entry corresponding to said neighbor is retrieved and the therein contained preferred interface identifier (which is NOT a MAC address) is passed to the `UIPBUF_ATTR_INTERFACE_ID` attribute. As you undoubtedly know by now, the 6LoWPAN adaptation layer (see `os/net/ipv6/sicslowpan.c`) shall copy this attribute to the corresponding `packetbuf` attribute (i.e., `PACKETBUF_ATTR_INTERFACE_ID`) further down the line, after which the twofaced MAC protocol eventually calls a twofaced-rf radio driver abstraction's `set_value()` function passing the `RADIO_PARAM_SEL_IF_ID` radio parameter and a value copied from the `PACKETBUF_ATTR_INTERFACE_ID` attribute value (see `send()` in `examples/twofaced/net/mac/twofaced-mac/twofaced-mac.c` and `send_one_packet()` in `examples/twofaced/net/mac/twofaced-mac/twofaced-mac-output.c`) in order to change the currently selected interface.

> ***Note:*** *the [access rules for Contiki-NG packet buffers] (amongst which `uip_buf` and `packetbuf`) state that `uip_buf` may only be accessed from the 6LoWPAN adaptation layer (see `os/net/ipv6/sicslowpan.c`) and above, while the `packetbuf` may only be accessed from the 6LoWPAN adaptation layer and below.*

```
send_packet:
  if(nbr) {
    linkaddr = uip_ds6_nbr_get_ll(nbr);
    if(!uipbuf_is_attr_flag(UIPBUF_ATTR_FLAGS_ALL_INTERFACES)) {
      const struct link_stats *stats = link_stats_from_lladdr((linkaddr_t *)linkaddr);
      if(stats != NULL) {
        ...
        uipbuf_set_attr(UIPBUF_ATTR_INTERFACE_ID, stats->pref_if_id);
      }
    }
  } else {
    linkaddr = NULL;
  }
  ...
  tcpip_output(linkaddr);
```

Simulation

Cooja

There are generally two ways to simulate experiments using the Cooja network simulator, i.e., by running Cooja in a Docker container or running it natively on Linux (tested for Ubuntu 18.04 LTS and should work on 20.04 LTS). The downside of running Cooja natively is that not all node types are supported in a 64-bit version of Linux, which is most likely what you'll be running. While future implementations of this example will most likely rely on running Cooja natively, for now, the Docker approach suffices. Before you can run Cooja through Docker however, you must first go through the installation process.

The installation process (for Ubuntu) starts by making sure there are no older versions of Docker installed. Next, it is recommended to install Docker through its repository as explained here. Following the installation of Docker, you must first make sure your user is added to the `docker` group and rebooting as follows:

```
$ sudo usermod -a -G docker <user>
$ sudo reboot
```

Then, you can pull the latest Docker Contiki-NG image:

```
$ docker pull contiker/contiki-ng
```

Finally you must append the following lines to `~/.bashrc`:

```
export CNG_PATH="$PATH:$HOME/contiki-ng"
alias contiker="docker run --privileged --sysctl net.ipv6.conf.all.disable_ipv6=0 --mount type=bind,source=$CNG_PATH,destination=$PATH:$HOME/contiki-ng -e DISPLAY=$DISPLAY -v /tmp/.X11-unix:/tmp/.X11-unix -v /dev/bus/usb:/dev/bus/usb -ti contiker/contiki-ng"
```

and run the following command:

```
$ exec bash
```

That's normally all the steps required to start with Cooja. However, since we've made some modifications to Cooja for dual-interface support, this is where you change out the default Cooja implementation included with Contiki-NG for our implementation. You can do this by means of some Git submodule hocus pocus:

```
cd ~/contiki-ng
git config --file=.gitmodules submodule.tools/cooja.url https://github.com/tinstructor/cooja.git
git config --file=.gitmodules submodule.tools/cooja.branch dripl
git submodule sync
git submodule update --init --recursive
cd tools/cooja/
git fetch
git checkout dripl
git checkout f39cfd248f379b8fdec36dd2d09d7ca00850a9e6
cd ../..
```

> ***Note:*** *It's probably a good idea to make a custom command to perform this task repeatedly.*

If you want to change back to the default Cooja implementation, you can run:

```
cd ~/contiki-ng
rm -rf tools/cooja
git checkout HEAD -- .gitmodules
git submodule update --init --recursive
git submodule sync
git submodule update --init --recursive
cd tools/cooja/
git remote prune origin
```

```
git branch -D dripl
cd ../..
```

> ***Note:** It's probably a good idea to make a custom command to perform this task repeatedly.*

You are now ready to start simulating with Cooja! Running Cooja in a Docker container is as easy as typing:

```
$ contiker cooja
```

which creates a Docker container and opens Cooja. When exiting Cooja, the docker container will halt too. It will, however, not be removed and can be restarted if you wish. This also means that it takes up disk space though! Since the purpose of the Docker container is merely to provide all dependencies required to simulate and compile examples written for Contiki-NG, who's codebase is attached to a Docker container (that is, if it's created with the `contiker` command) as a bind mount, and nothing more, it makes no sense to leave halted containers in memory. Removing halted Docker containers can be done as follows:

```
$ docker container prune
```

> ***Note:** A list of useful Docker commands is provided in [this cheatsheet](#).*

Alternatively, and we recommend this approach, you can manually create a Docker container and run Cooja. This allows you to perform some cleaning operations prior to switching to a different simulation config (more about that later). You can do this as follows:

```
$ contiker
...
user@83dab95d6216:~/contiki-ng$ cd tools/cooja/
user@83dab95d6216:~/contiki-ng/tools/cooja$ ant clean
...
user@83dab95d6216:~/contiki-ng/tools/cooja$ ant run
```

Anyhow, when you've opened a Docker container and started Cooja, you can open the provided simulation file(s) by clicking on `file > Open simulation > Open and Reconfigure` (from the tab menus in the top-left corner) as shown in the following figure:

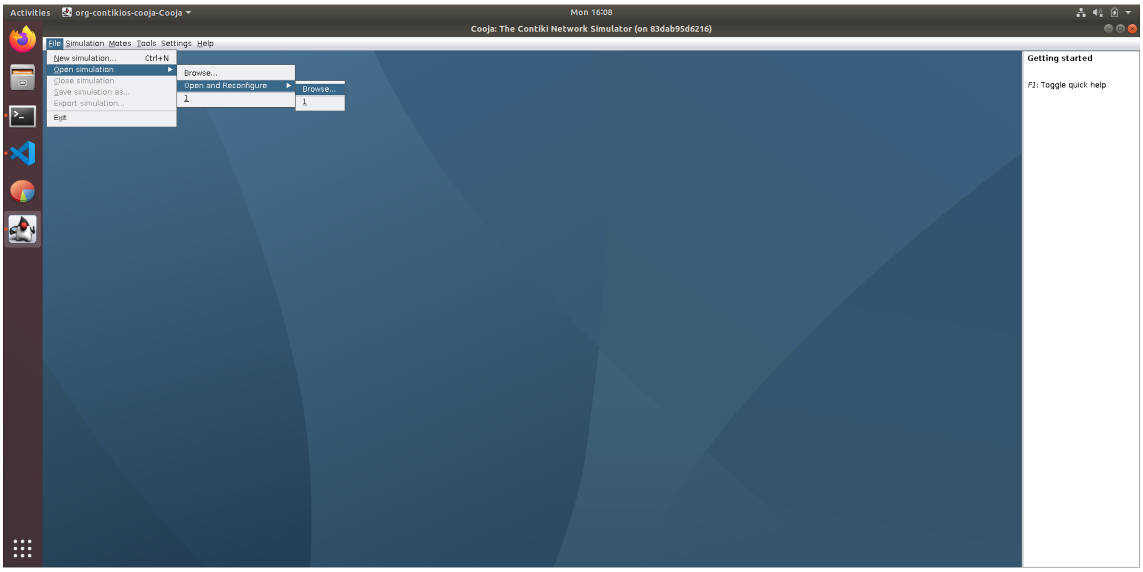

Next, browse to the `twofaced` example directory as follows:

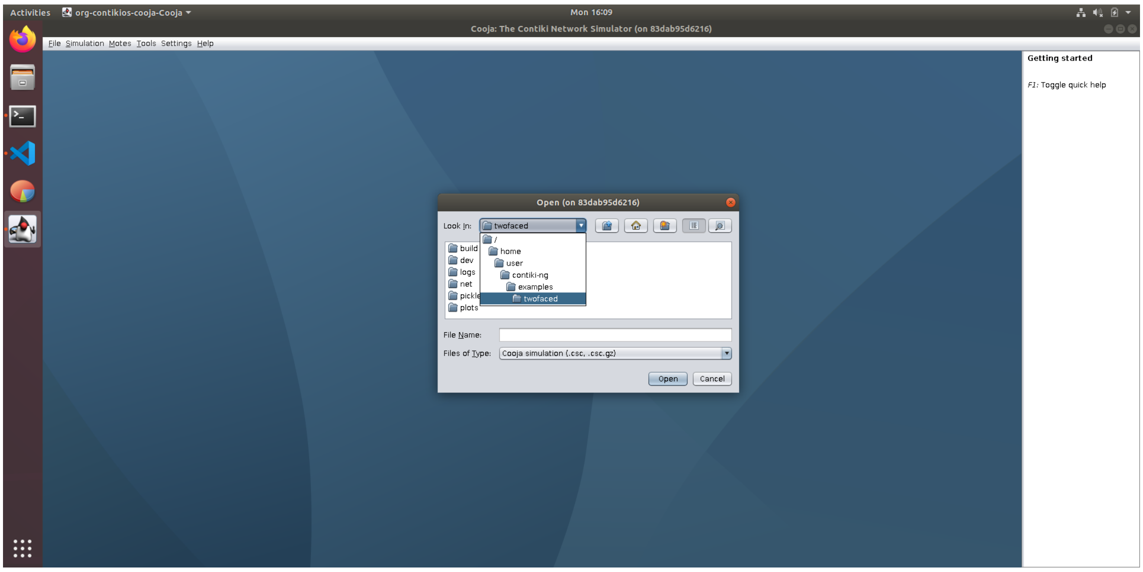

Then select the Cooja simulation (`.csc`) file named `small_grid.csc` (`large_grid.csc` works the same but it is much slower because it involves way more nodes). The following window will appear wherein the `LogisticLoss` radio medium is already selected and the box for generating a new random seed upon reload is already ticked (although you shouln't really trust this box, but more about how we solve that later). Al you have to do is click `create` .

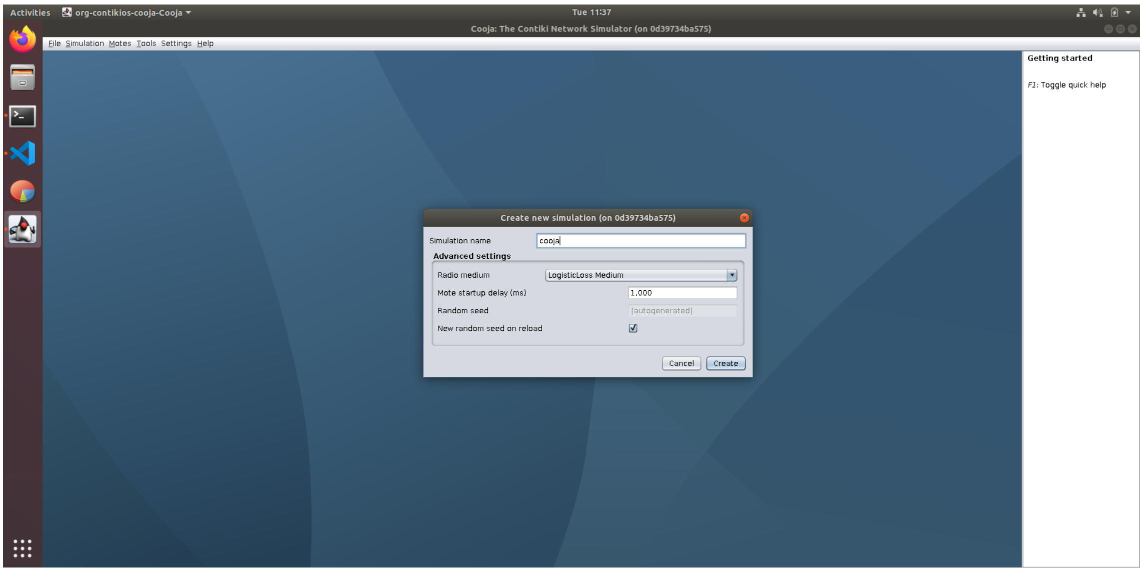

Next, Cooja will ask to (re-)compile the latest binaries of the selected firmware (which you may have altered outside the Docker container in the meantime) for each type of "mote" present in the simulation file. The only thing you need to do right now is press `compile` and `create` (in that order) for every "mote" type pop-up. When you have modified the twofaced project (and possible the sim file) for single-interfaced operation, additional steps are required (more on that later).

> ***Note:*** *another pop-up may open first asking to select the correct mote type from a drop-down list. Just ignore this and click on* `ok`*.*

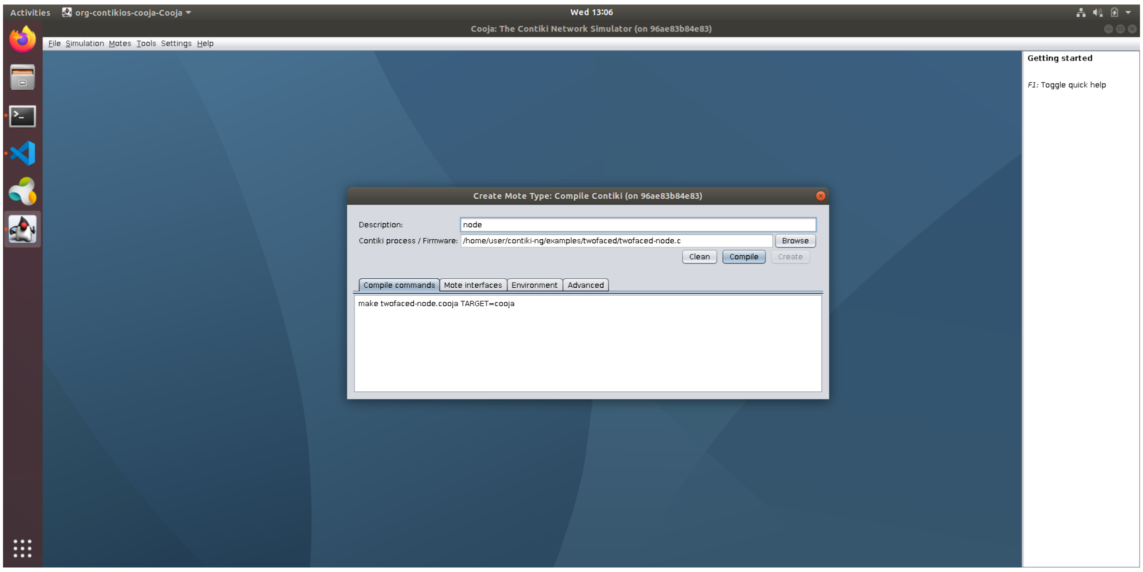

Upon pressing the `create` button for the last "mote" type pop-up (of which there'll be 3 in our case, because there'll also be a coojammer node, see `examples/coojammer/coojammer-node.c`), the Cooja simulation window will open. This window contains a number of panels, among which the `Simulation control` and `Mote output` panel. The `Simulation control` panel allows you to start, pauze, stop, and

reload the current simulation and gives an indication of the simulated time and the simulation speed (i.e., how much faster or slower the simulation is w.r.t. system speed). Press the `start` button, wait for say 15 simulation seconds, and hit `pauze`. Note that the `Mote output` panel contains the terminal output for each node in the simulation. Different nodes can be distinguished by means of their color in the `Mote output` panel and via their Node ID. The `Mote output` panel also allows you to save the combined terminal output (of all nodes in the simulation) to a separate `.txt` file (or append to an existing `.txt` file) for further (external) processing.

> ***Note:*** *in order to save the serial output of all motes (i.e., the contents of the* `Mote output` *panel) to a logfile, al you got to do is go to the* `File` *dropdown menu in the* `Mote output` *panel and click on* `Save to file`

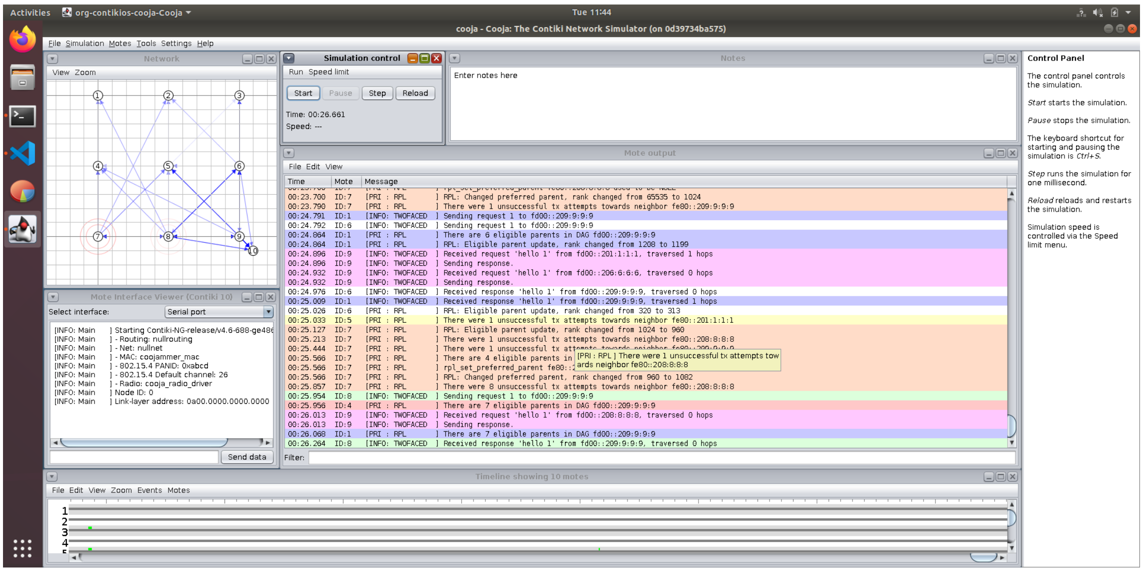

Experiments

In the accompanying paper, we talked about 4 different simulation setups. By default, the twofaced project is configured such that the simulated network runs DRiPLOF. If you've ever made changes in the future (as we will explain shortly) and want to go back to DRiPLOF, you need to uncomment the following line in `examples/twofaced/project-conf.h` (which is already the case by default):

```
#define LINK_STATS_CONF_PREF_IF_METRIC_ONLY 1
```

Besides, you also want to make sure the root node is disseminating the correct OCP and that the non-root nodes support the corresponding OF by making sure the following lines are set correct in `examples/twofaced/project-conf.h` (which is already the case by default):

```
#define RPL_CONF_OF_OCP RPL_OCP_DRIPLOF
#define RPL_CONF_SUPPORTED_OFS { &rpl_driplof }
```

Finally, in `examples/twofaced/Makefile`, you must make sure the MAKE_MAC variable is set up correctly as follows (which is already the case by default):

```
MAKE_MAC = MAKE_MAC_TWOFACED
```

All other stuff should be left untouched, except maybe the following macros in `examples/twofaced/project-conf.h` . If you want to have a better understanding of how everything works, it could be advantageous to set the debug level of different modules to something other than `LOG_LEVEL_NONE` , which is the default, and means that the corresponding module will not print debug statements to the terminal (which would show up in Cooja's `Mote output` panel). However, to properly simulate a network for parsing and visualization purposes, it is best to leave it as is, since otherwise the debug statements may overflow the Cooja log buffer, causing the log parsing to fail (more about that later).

```
#define LOG_CONF_LEVEL_APP      LOG_LEVEL_INFO
// #define LOG_CONF_LEVEL_RPL      LOG_LEVEL_DBG
// #define LOG_CONF_LEVEL_TCPIP    LOG_LEVEL_DBG
// #define LOG_CONF_LEVEL_IPV6     LOG_LEVEL_DBG
// #define LOG_CONF_LEVEL_6LOWPAN  LOG_LEVEL_DBG
// #define LOG_CONF_LEVEL_MAC      LOG_LEVEL_DBG
// #define LOG_CONF_LEVEL_FRAMER   LOG_LEVEL_DBG
```

Before you start a simulation, it is probably handy to make the simulation stop after a predetermined amount of time. As the log parsing scripts expect a simulation time of 20 minutes (i.e., 1200 seconds) by default (this can be changed easily, more on that later) you must let each simulation run for at least 20 minutes (because the log parsing script automatically cuts all lines after said time such that all logs contain information for the exact same amount of time, more on that later). This is easily achieved by (ab)using Cooja simulation scripts. In order to open the Cooja simulation script editor, do the following:

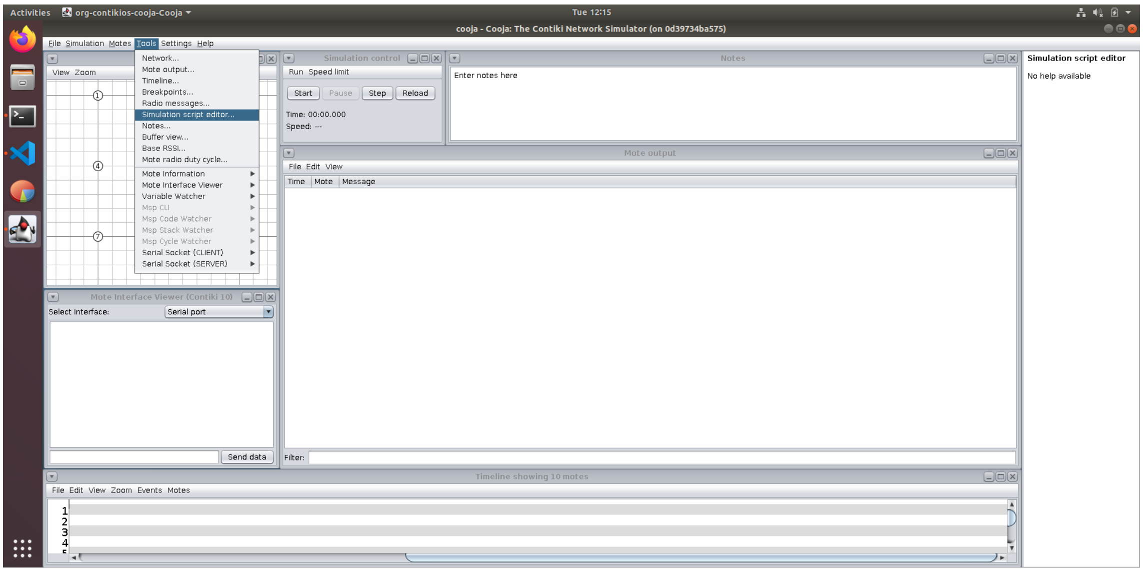

A new window will open in the Cooja simulator. In this window you can put certain Javascript commands that the Cooja simulator understands. For example, you can put a `TIMEOUT` statement and specify a certain amount of milliseconds. If the simulation is still running after said amount of milliseconds, Cooja will automatically pause and declare a "failed test" (which really has no meaning because it does nothing). As such we can use such statement to automate things a bit:

> ***Note:*** *the Cooja simulation scripts are much more powerful than the simple example we just gave. As a matter of fact, they can also be used to write serial input to the terminal of a simulated mote for example. We have used to this to perform our jamming simulations. More specifically, a jammer node (see* `examples/coojammer` *) is able to perform certain actions upon receiving certain commands on its serial interface. Then, with the (contents of the) simulation scripts called* `examples/twofaced/small_grid.js` *(for the small grid simulation) and* `examples/twofaced/large_grid.js` *(for the large grid simulation) one can make the jamming node send out corrupted data packets over a certain interface, for a given amount of time, and at a certain point in (simulation) time.*

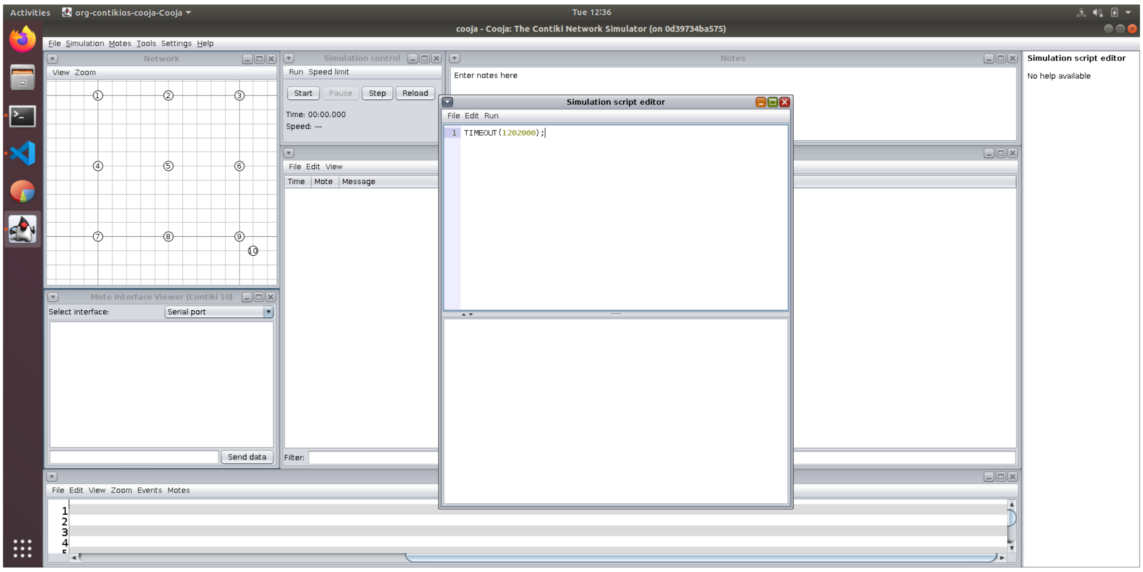

Next, click on `Run > Activate` in the `Simulation script editor` window. Then, click on `Reload simulation > Reload with new random seed` in the top left corner, prior to every simulation run, just to make sure:

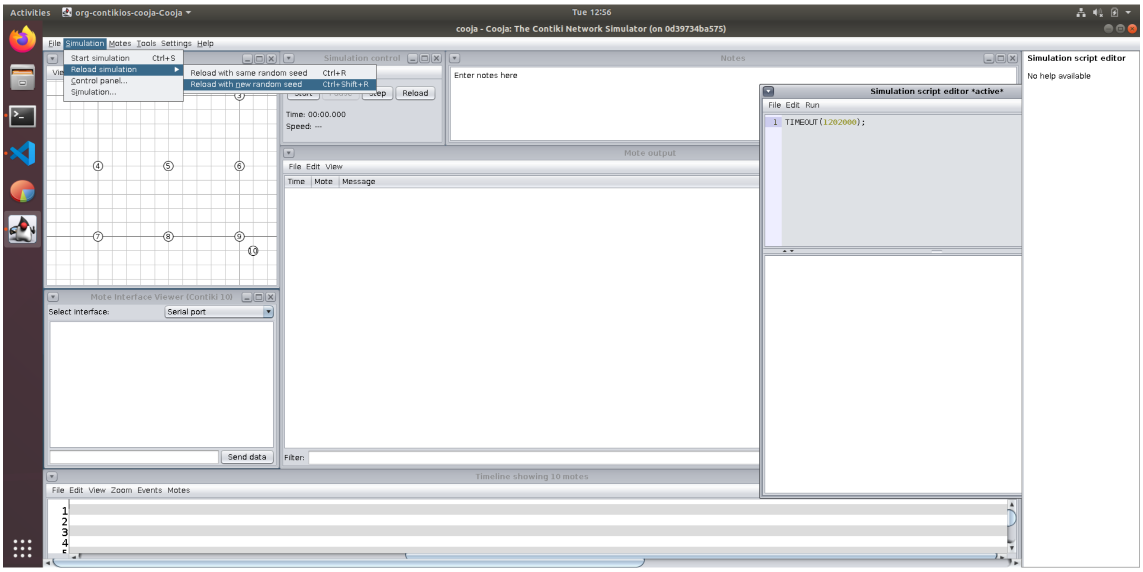

If you like, you can set a simulation speed limit in the `Simulation control` window. The speed limit you choose is pretty important to make sure the simulation doesn't run itself into the ground, especially when simulating large networks. Therefore we recommend to leave this at 100% or 200% when running the `large_grid.csc` simulation config and for the `small_grid` simulation config, it should probably not be set higher than 400%. Anyhow, you can click on `start` in the `Simulation control` window and come back after 20 minutes (or less if the sim speed limit was set to higher than 100% and the simulation was actually able to run that fast). If everything went well (which it should've), the simulation has paused somewhere close after the 20-minute mark. You can now save the contents of the `Mote output` window to a logfile and store it in the appropriate location (which is `examples/twofaced/logs/`) as follows:

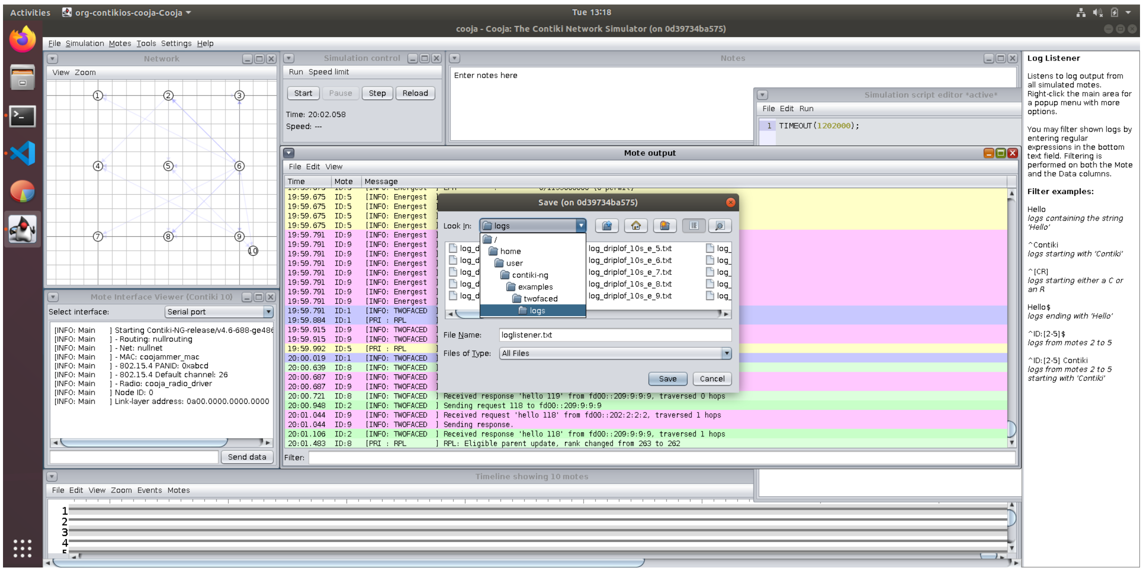

You can now repeat the process of reloading the simulation with a random seed, hitting start, waiting for the simulation to halt after 20 minutes, and saving the contents of the mote terminals to a logfile for as many times as you want (for the given simulation configuration). Make sure to use appropriate names for the logfiles (see `examples/twofaced/multilogparser.py`).

> ***Note:*** *it may happen that Cooja refuses to reload or starts to behave erratically because of some memory issue. This typically happens after a number of (long) simulation runs. Luckily Cooja generally warns that it's about to run out of memory, and you should not ignore this warning. Instead you should follow the same process as for switching between simulation configurations (which will be explained shortly) but just keep the same configuration.*

Then, let's say you want to change the simulation configuration such that the network runs POOF. In that case, close the Cooja simulator **without saving the current simulation**. In the terminal window from which you have started Cooja, type the following:

```
...
user@83dab95d6216:~/contiki-ng/tools/cooja$ ant clean
...
user@83dab95d6216:~/contiki-ng/tools/cooja$ exit
```

Next, make sure that the next time you open a Cooja docker container, a new one is created by cleaning up the old ones with `docker container prune`:

```
$ docker container prune
WARNING! This will remove all stopped containers.
Are you sure you want to continue? [y/N] y
Deleted Containers:
6236ec71c056dac46c22749d15aee8a0d446e144c1c96c6c769cb91cd7fc88af

Total reclaimed space: 31B
```

Then, navigate to `examples/twofaced/project-conf.h` and comment out the following line:

```
// #define LINK_STATS_CONF_PREF_IF_METRIC_ONLY 1
```

Also modify the following lines in `examples/twofaced/project-conf.h`:

```
#define RPL_CONF_OF_OCP RPL_OCP_POOF
#define RPL_CONF_SUPPORTED_OFS { &rpl_poof }
```

And leave `examples/twofaced/Makefile` as-is. Now you can, once again, start Cooja manually from a terminal window as follows:

```
$ contiker
...
user@83dab95d6216:~/contiki-ng$ cd tools/cooja/
user@83dab95d6216:~/contiki-ng/tools/cooja$ ant run
```

The remaining steps of the simulation process are identical for POOF and DRiPLOF. Then, let's say you want to switch to MRHOF, which is single-interface, and you want to simulate nodes with high-rate / short-range 2.4 GHz interfaces only. First, after running the last POOF simulation and saving the latest log file, you repeat the simulation exit and cleanup process we just described in detail. Then you want to navigate to `examples/twofaced/project-conf.h` once again and change the following lines only (the other line we previously changed doesn't matter):

```
#define RPL_CONF_OF_OCP RPL_OCP_MRHOF
#define RPL_CONF_SUPPORTED_OFS { &rpl_mrhof }
```

Next, go to `examples/twofaced/Makefile` and change the MAC protocol to be used by modifying the following line:

```
MAKE_MAC = MAKE_MAC_CSMA
```

Now, start Cooja manually as you did before (make sure you have executed `ant clean` when you performed the last simulation exit and cleanup process because this is important):

```
$ contiker
...
user@83dab95d6216:~/contiki-ng$ cd tools/cooja/
user@83dab95d6216:~/contiki-ng/tools/cooja$ ant run
```

Then, after selection the appropriate simulation file from `examples/twofaced` (again, via `file > Open simulation > Open and Reconfigure > Browse`), you will end up at the create and compile step again (for every mote type). However, this time, (**for the `node` and `root` mote types only, leave the `jammer` mote type as-is**) you first navigate to the `Mote interfaces` tab and untick the box next to `Twofaced Radio Interface`, which will cause Cooja to **not** attach a low-rate / long-range 868 MHz interface to every node (the cooja radio driver `arch/platform/cooja/dev/cooja-radio.c` is already set up correctly by modifying `examples/twofaced/Makefile`). As alway, press `clean`, `compile`, and `create`, in that order.

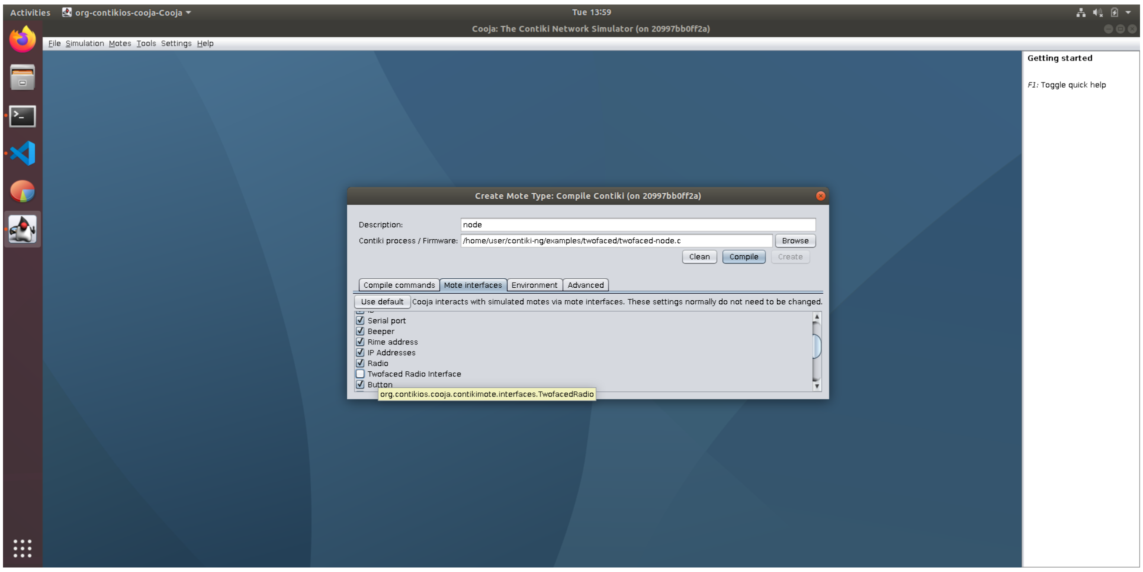

The remaining simulation steps are the same as before. Finally, after properly performing a simulation exit and cleanup (after running all high-rate MRHOF sims), stuff gets a litlle tricky, **so here is where you should really start paying attention** or otherwise you will get bad simulation results and you might falsely accuse us of forgery because you did something wrong yourself.

For example, let's say you now want to simulate a single-interface networking running MRHOF but with low-rate interfaces only. If the previous simulation was also MRHOF but with high-rate interfaces, you don't need to make any changes to `examples/twofaced/project-conf.h` nor `examples/twofaced/Makefile`. Otherwise make the same changes as in the previous simulation configuration. Anyhow, when simulating a small grid of nodes **we now need to modify `examples/twofaced/small_grid.csc` prior to starting Cooja manually** (the same is true for `examples/twofaced/large_grid.csc` when simulating a large network). What we need to do is copy all radio medium variables belonging to the 868 MHz radio to the 2400 GHz radio, effectively turning the 2400 GHz radio into an 868 MHz radio as far as Cooja is concerned (in terms of its performance over the air):

```
    <radiomedium>
      org.contikios.cooja.radiomediums.LogisticLoss
      <transmitting_range_2400>196.3033798012039</transmitting_range_2400>
      <transmitting_range_868>196.3033798012039</transmitting_range_868>
      <success_ratio_tx>1.0</success_ratio_tx>
      <reference_distance>1.0</reference_distance>
```

```
        <path_loss_ref_dist_2400>31.212166689578513</path_loss_ref_dist_2400>
        <path_loss_ref_dist_868>31.212166689578513</path_loss_ref_dist_868>
        <rssi_inflection_point>-92.0</rssi_inflection_point>
        <path_loss_exponent_2400>3.0</path_loss_exponent_2400>
        <path_loss_exponent_868>3.0</path_loss_exponent_868>
        <awgn_sigma_2400>5.0</awgn_sigma_2400>
        <awgn_sigma_868>5.0</awgn_sigma_868>
        <enable_time_variation>false</enable_time_variation>
      </radiomedium>
```

Next, make sure all 2.4 GHz interfaces in the network (i.e., of every node) have their bitrate set to that of the 868 MHz interface by means of a `Ctrl + F` + `replace all` operation (make sure to hit save):

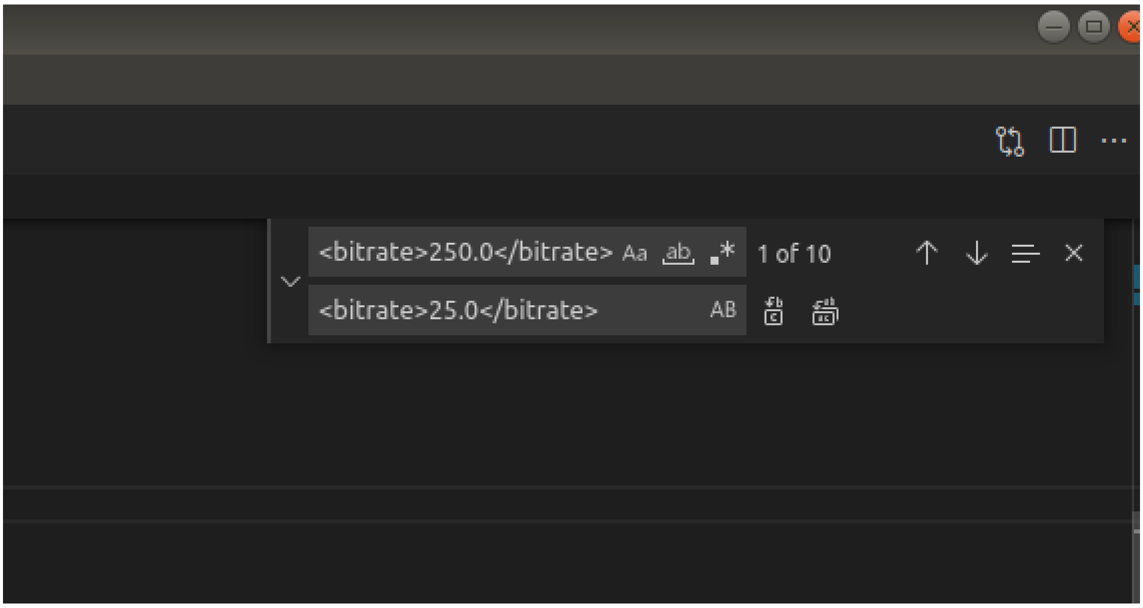

Now, when the Cooja simulator loads the simulation file (e.g., `examples/twofaced/small_grid.csc`), the radio medium instance will use the variable stored in the sim file to overwrite the variables it assigns by default, effectively tricking Cooja into thinking that its 2.4 GHz radio is an 868 MHz radio (actually, it's not even a trick because the behavior will be identical). Now, you can start the Cooja simulator as you did before and perform the same steps as with high-rate MRHOF.

> ***Note:*** *if you are using the simulation script editor to run the contents of* `examples/twofaced/small_grid.js` *for example (i.e., when simulating a jamming scenario), make sure to instruct the jammer node to jam on the correct interface. For the single-interface simulation configs you want to jam on the interface with* `id = 1`*, otherwise on the interface with* `id = 2`

Log Parsing & Visualization

For the sake of brevity, I shall only explain how to use the `twofaced` example folder structure and Python scripts to visualize simulation data. The parsing and visualization mechanism is divided into 3 parts, each with a corresponding Python script, of which you'll only need to use 2 (because 1 is automated). The `examples/twofaced/logparser.py` script operates on a single logfile, parses it, and

serializes a whole slew of statistics to Python pickles (which is a fast, relatively small footprint, but non-human-readable serialization format that works way faster than the traditionally used `.csv` files). The `examples/twofaced/logparser.py` script takes a number of arguments when called. Since you have placed the simulation logs in the appropriate folder (i.e., `examples/twofaced/logs`) and have given them appropriate names (no need to panick, you can still change this), you don't really need to use this script. Instead, usage of this script on every logfile is automated by running `examples/twofaced/multilogparser.py`. This script works as follows. First you set the duration of any given simulation experiment (you can verify this by looking at the last timestamps in the logfiles) in seconds by means of the `experiment_duration_seconds` variable, next you specify the transmit power with which all radios send their signals, then you specify an array of partial command strings. Finally, the `examples/twofaced/multilogparser.py` script loops over this array and repeatedly calls the `logparser.py` script by forming the next complete command string and executing it.

> ***Note:*** *for simplicity's sake we assumed that all radios transmit with the same power (largely because the module that allows us to track energy consumption doesn't distinguish between radios), and so it is not possible to specify a different transmit power for both radios.*

```
experiment_duration_seconds = 1200
tx_power_dbm = 0

logparser_cmds = []
logparser_cmds += ["log_poof_10s_e_%u.txt POOF" % i for i in range(1,11)]
logparser_cmds += ["log_driplof_10s_e_%u.txt DRiPLOF" % i for i in range(1,11)]
logparser_cmds += ["log_mrhof_ht_10s_e_%u.txt MRHOF_HT" % i for i in range(1,11)]
logparser_cmds += ["log_mrhof_lt_10s_e_%u.txt MRHOF_LT" % i for i in range(1,11)]

# ...
for logparser_cmd in logparser_cmds:
    # ...
    logparser_cmd = "python3 logparser.py ./logs/" + logparser_cmd + " %d -a --
log_cutoff %d" % (tx_power_dbm, experiment_duration_seconds)
    # ...
    py_shell =
subprocess.Popen(shlex.split(logparser_cmd),stdout=PIPE,stderr=PIPE,universal_newlines=[illegible]

# ...
```

Next, if you are sure that the simulation logs have the correct file names, and you have specified the correct range of logs to be parsed, run the `examples/twofaced/multilogparser.py` script (from the `examples/twofaced` directory) as follows:

```
~/contiki-ng/examples/twofaced$ python3 multilogparser.py
```

> ***Note:*** *if, for some reason, you have stored a range of simulation logs called log_poof_10s_e_1.txt through log_poof_10s_e_10.txt but you only want to parse the last 5, you would modify the corresponding range to* `logparser_cmds += ["log_poof_10s_e_%u.txt POOF" % i for i in range(6,11)]`

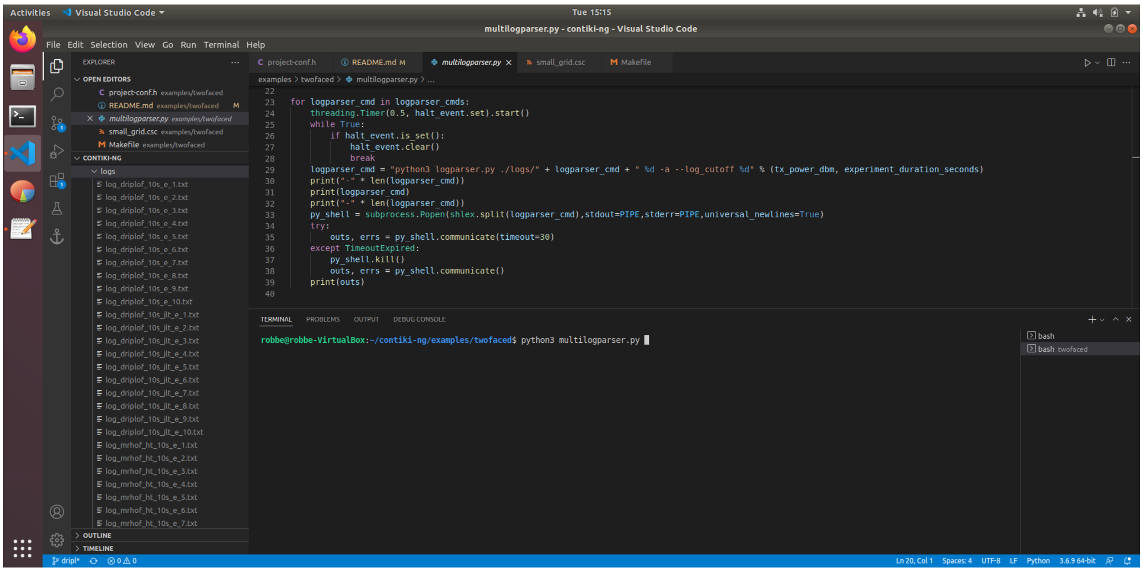

This will print every parsing command (i.e., every call to the `logparser.py` script), followed by the terminal output of that script. This terminal output contains some general statistics that you may or may not find useful:

```
robbe@robbe-VirtualBox:~/contiki-ng/examples/twofaced$ python3 multilogparser.py
----------------------------------------------------------------------------
python3 logparser.py ./logs/log_poof_10s_e_1.txt POOF 0 -a --log_cutoff 1200
----------------------------------------------------------------------------
The average end-to-end delay for upward data traffic:   0:00:00.528300
The average end-to-end delay for downward data traffic: 0:00:00.433448
The average round-trip-time for data traffic:           0:00:00.942809
The PDR at the root:                                    94.887%
The PDR for packets sent (down) by the root:            98.094%
Total number of parent changes:                         354
Average number of parent changes per node:              14.750
Average seconds spent as orphan since root startup:     6.064
The global twa number of hops:                          2.908
The average of the per-node twa number of hops:         2.910
The global twa parent set size:                         4.809
The average of the per-node twa parent set sizes:       4.617
The global twa node rank:                               2142.919
The average of the per-node twa ranks:                  2143.395
The total number of retransmissions:                    19156
The average of the per-node tx energy (in mJ):          38.210

----------------------------------------------------------------------------
python3 logparser.py ./logs/log_poof_10s_e_2.txt POOF 0 -a --log_cutoff 1200
```

When `multilogparser.py` completes and terminates, you will find a number of pickles in `examples/twofaced/pickles`. These pickles are a serialized form of a set of Python dictionaries that contain all the simulation stats of the logfiles you instructed `multilogparser.py` to parse. Since the visualization script (more info later) takes all the data stored in these pickles, it is important that you make a conscious decision when specifying which logs to parse in `examples/twofaced/multilogparser.py`. More specifically, you should only specify those logfiles that are supposed to be visualized in the same graphs down the line. For example, prior to wanting to visualize a jamming scenario, you should remove all pickles from

`examples/twofaced/pickles` and run `examples/twofaced/multilogparser.py` again for the logfiles that were generated during the jamming scenario (or part of them).

Anyhow, the next step is to generate graphs from the pickled simulation data by running the `examples/twofaced/pickleparser.py` script. By default, this will produce a number of graphs / plots in `examples/twofaced/plots` in `.pdf` format (because we required a vector-based format for inclusion into a paper). However, one could easily generate pngs instead by means of an optional argument:

```
~/contiki-ng/examples/twofaced$ python3 pickleparser.py -e png
```

You can also specify a string that is to be appended to the default plot names as follows:

```
~/contiki-ng/examples/twofaced$ python3 pickleparser.py -e png -s jamming
```

This allows, for example, to give the plots generated from the pickles that represent the stats of a different simulation scenario to have a different name such that they don't overwrite the plots generated from another scenario.

> *More information available upon request.*

Uncrustify

Before submitting merge requests of any kind, please make sure your code (and file-naming) adheres to [the code style guidelines of Contiki-NG](). A good way to rectify slopily written source code is to use `uncrustify` (a tool) with some style scripts provided by Contiki-NG. First off, install Uncrustify as follows:

```
$ sudo apt-get install uncrustify
```

Now, before I explain the general usage of the provided scripts, you're going to define some custom Linux commands. More specifically, let's create two commands that'll allow you to either retrieve the recommended style changes to a file (`checkcrust`) or automatically apply these changes on disk (`fixcrust`). For safety's sake, I've limited the amount of arguments the `fixcrust` command accepts to just one file (or its location) instead of the space-delimited list of files (or their locations) it normally accepts (that is, the underlying script does). The `checkcrust` command only accepts a single argument (i.e., a code file or its location) as well. However, this is not a safety feature, but merely a limitation of the underlying script provided by Contiki-NG. Anyhow, we first create two files in `~/bin/` :

```
$ cd ~/bin/
$ touch checkcrust fixcrust
```

Next, add the following lines to `checkcrust` :

```
#!/bin/bash
~/contiki-ng/tools/code-style/uncrustify-check-style.sh $1 2>&1
```

and the following lines to `fixcrust` :

```
#!/bin/bash
~/contiki-ng/tools/code-style/uncrustify-fix-style.sh $1 2>&1
```

Then, we give these files all rights imaginable because, once again, we're only partially pedantic about good practices and sometimes (read: often) we only pretend to care:

```
$ cd ~/bin/
$ sudo chmod 777 checkcrust fixcrust
```

Finally, restart your shell or execute the following convenient command so that the `PATH` variable is updated and now also points to `~/bin/` :

```
$ exec bash
```

In the following example we added some innapropriate indentation on line 50 and used the `checkcrust` command to see if the supplied code file was properly formatted (which of course it wasn't). Upon seeying that the only flaw (identified by uncrustify, that is) is said indentation, we used the `fixcrust` command to rectify this error automatically.

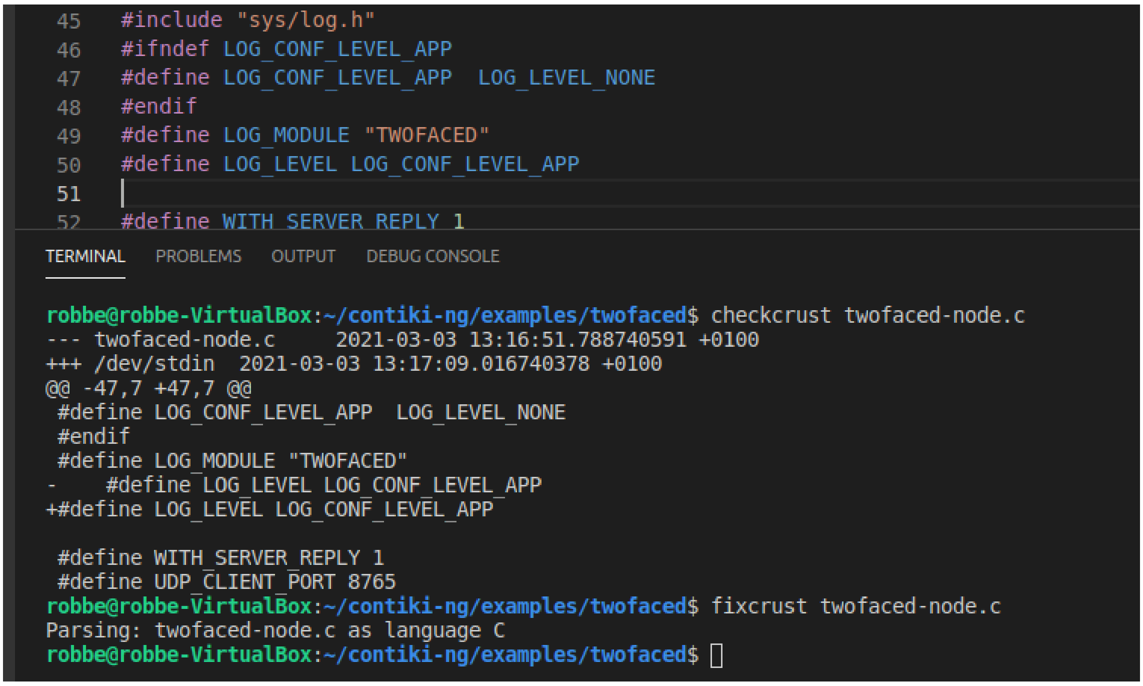

> ***Note:*** *As stated in [the official docs](), uncrustify is not some magic silver bullet and will sometimes misformat your code! Be aware of this! As such, it's probably better in most cases to only use `checkcrust` instead of `fixcrust` .*

Recommended Resources

- **The Contiki-NG Wiki**: It is generally advisable to start out here. However, the documentation is really basic and leaves much to be desired, especially if you want to do more than just write applications on top of existing functionality.
- **Practical Contiki-NG** *by Agus Kurniawan*: After you've gone through the Wiki you'll have a lot of unanswered questions about how stuff actually works. This book can help you with a small part of those questions, but don't expect an in-depth explanation of anything PHY related. If you have a Shibboleth or Athens

account via your research institution, you can even download a digital copy for free, legally!

- **IEEE 802.15.4-2020**: The currently active PHY + MAC layer standard for 802.15.4 networks. Although this is the official standard, many developers seem to have a total disregard for certain aspects of it. Especially on the Sub-GHz PHY layers, there seems to be a lot of confusion as to what is actually standardised and what is not. The fact that IEEE standards are not publicly available and otherwise very expensive to obtain doesn't help this confusion either.
- **Jump Start Git** *by Shaumik Daityari*: As mentioned before, if you don't speak Git, this book is where you might want to start your journey.
- **C in a Nutshell** *by Peter Prinz & Tony Crawford*: Although C is an incredibly forgiving language when it comes to getting what you want out of it (hence the abundance of terribly written but otherwise "functional" code), some parts of our source code contain more advanced features and contstructs from the C99 and later C11 specification. We make no mistake about it, seasoned embedded developers would probably have a heart-attack looking at parts of our code, but the important thing to remember is that we're well on our way to write better source code and this book is what's getting us there.
- **The Renode docs**: pretty elaborate documentation of Renode and the functionality it provides. Is sufficient for most use cases but lacks when it comes to scenarios requiring heavy modification, let alone the creation of, Renode source files.
- **The Java Programming Masterclass** *by Tim Buchalka*: want to learn modern Java programming with minimal to no experience in Object-Oriented Programming (OOP)? Just looking for a refresher on OOP best-practices and / or the Java programming language because it has been a while? Whatever your skill- and / or comfort level, this beautifully structured online course will get you there in no-time (provided you are willing to put in the work). Happy hunting! (This course is especially useful if you want to understand / modify the Cooja project)

Contact and Affiliation

Robbe Elsas

email: robbe.elsas@ugent.be

affiliation: IDLab Ghent University - imec

Licensing

Unless explicitly noted otherwise, all files belonging to this example are licensed under a BSD 3-Clause license:

> *Copyright (c) 2021, Ghent University. All rights reserved.*
>
> *Redistribution and use in source and binary forms, with or without modification, are permitted provided that the following conditions are met:*
>
> 1. *Redistributions of source code must retain the above copyright notice, this list of conditions and the following disclaimer.*
> 2. *Redistributions in binary form must reproduce the above copyright notice, this list of conditions and the following disclaimer in the documentation*

> *and/or other materials provided with the distribution.*
> 3. *Neither the name of the Institute nor the names of its contributors may be used to endorse or promote products derived from this software without specific prior written permission.*
>
> *THIS SOFTWARE IS PROVIDED BY THE INSTITUTE AND CONTRIBUTORS "AS IS" AND ANY EXPRESS OR IMPLIED WARRANTIES, INCLUDING, BUT NOT LIMITED TO, THE IMPLIED WARRANTIES OF MERCHANTABILITY AND FITNESS FOR A PARTICULAR PURPOSE ARE DISCLAIMED. IN NO EVENT SHALL THE INSTITUTE OR CONTRIBUTORS BE LIABLE FOR ANY DIRECT, INDIRECT, INCIDENTAL, SPECIAL, EXEMPLARY, OR CONSEQUENTIAL DAMAGES (INCLUDING, BUT NOT LIMITED TO, PROCUREMENT OF SUBSTITUTE GOODS OR SERVICES; LOSS OF USE, DATA, OR PROFITS; OR BUSINESS INTERRUPTION) HOWEVER CAUSED AND ON ANY THEORY OF LIABILITY, WHETHER IN CONTRACT, STRICT LIABILITY, OR TORT (INCLUDING NEGLIGENCE OR OTHERWISE) ARISING IN ANY WAY OUT OF THE USE OF THIS SOFTWARE, EVEN IF ADVISED OF THE POSSIBILITY OF SUCH DAMAGE.*
