# Supplementary material for: DRiPLOF: An RPL Extension for Multi-Interface Wireless Sensor Networks in Interference-Prone Environments
Source: Sensors (Basel). 2022 May 21;22(10):3906. doi: 10.3390/s22103906 (PMC9144939; doi:10.3390/s22103906)
Supplement: Supplementary file 1 [file sensors-22-03906-s001.zip › Supplementary/data/plots/baseline/pdf/e2e_box.pdf]

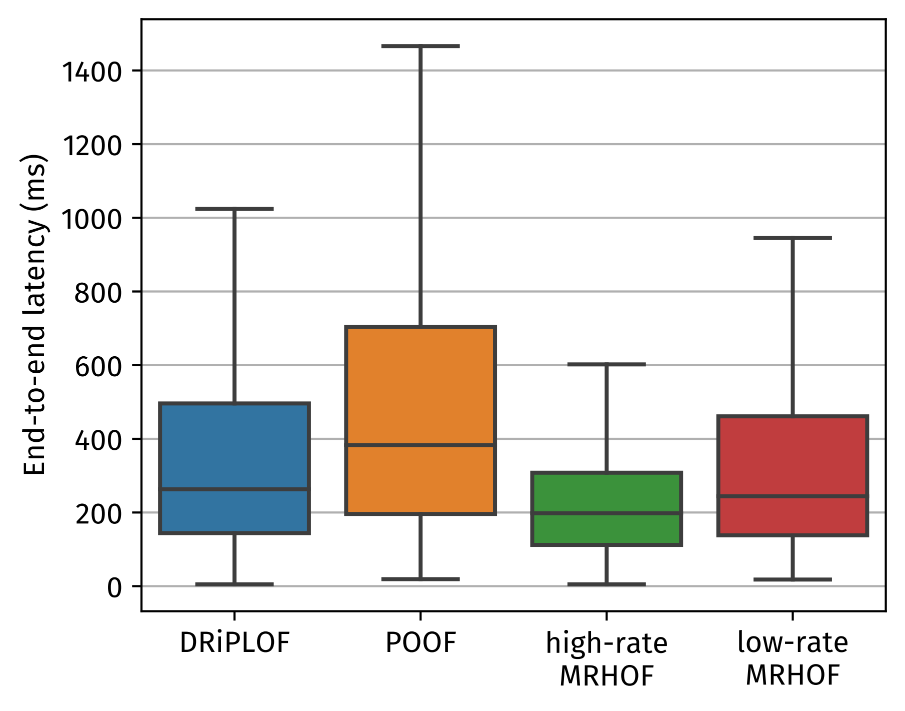
End-to-end latency (ms)
0
200
400
600
800
1000
1200
1400
DRiPLOF
POOF
high-rate MRHOF
low-rate MRHOF
