# Supplementary material for: DRiPLOF: An RPL Extension for Multi-Interface Wireless Sensor Networks in Interference-Prone Environments
Source: Sensors (Basel). 2022 May 21;22(10):3906. doi: 10.3390/s22103906 (PMC9144939; doi:10.3390/s22103906)
Supplement: Supplementary file 1 [file sensors-22-03906-s001.zip › Supplementary/data/plots/jamming/pdf/ecmj_scatter_median.pdf]

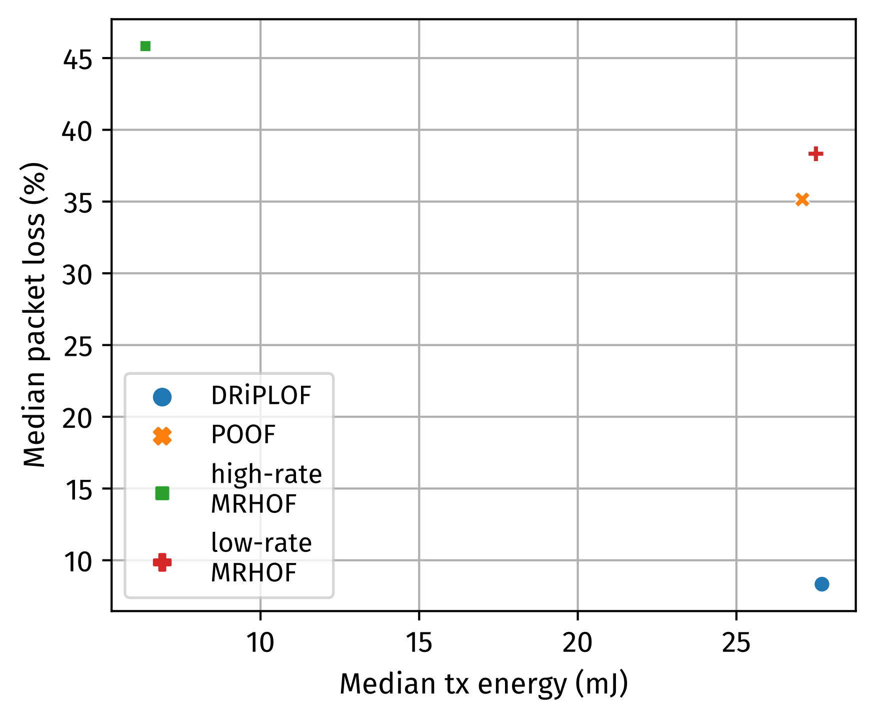

Median packet loss (%)
45
40
35
30
25
20
15
10
10
15
20
25
Median tx energy (mJ)
DRiPLOF
POOF
high-rate
MRHOF
low-rate
MRHOF
