# Supplementary material for: DRiPLOF: An RPL Extension for Multi-Interface Wireless Sensor Networks in Interference-Prone Environments
Source: Sensors (Basel). 2022 May 21;22(10):3906. doi: 10.3390/s22103906 (PMC9144939; doi:10.3390/s22103906)
Supplement: Supplementary file 1 [file sensors-22-03906-s001.zip › Supplementary/data/plots/jamming/pdf/ecmj_scatter_median_psim.pdf]

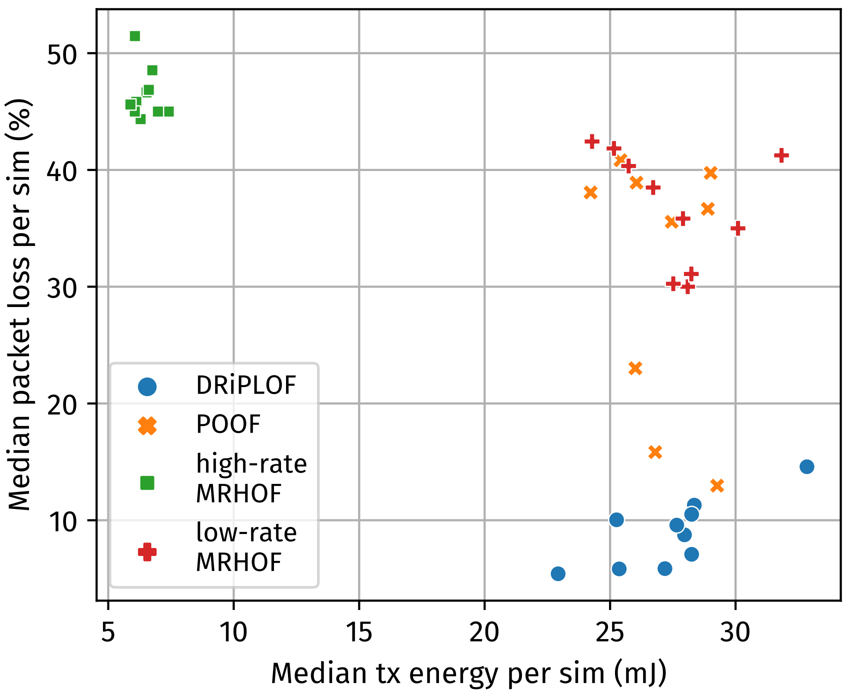

Median packet loss per sim (%)
50
40
30
20
10
5
10
15
20
25
30
Median tx energy per sim (mJ)
DRiPLOF
POOF
high-rate
MRHOF
low-rate
MRHOF
