# Supplementary material for: DRiPLOF: An RPL Extension for Multi-Interface Wireless Sensor Networks in Interference-Prone Environments
Source: Sensors (Basel). 2022 May 21;22(10):3906. doi: 10.3390/s22103906 (PMC9144939; doi:10.3390/s22103906)
Supplement: Supplementary file 1 [file sensors-22-03906-s001.zip › Supplementary/data/plots/jamming/pdf/psnpc_box.pdf]

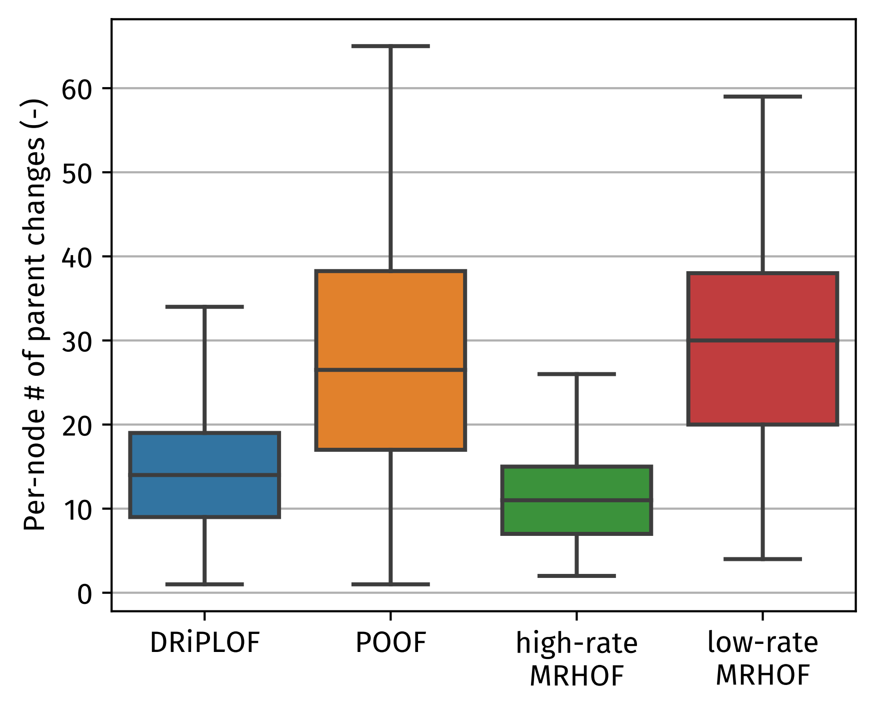
Per-node # of parent changes (-)
0
10
20
30
40
50
60
DRiPLOF
POOF
high-rate MRHOF
low-rate MRHOF
