# Supplementary material for: DRiPLOF: An RPL Extension for Multi-Interface Wireless Sensor Networks in Interference-Prone Environments
Source: Sensors (Basel). 2022 May 21;22(10):3906. doi: 10.3390/s22103906 (PMC9144939; doi:10.3390/s22103906)
Supplement: Supplementary file 1 [file sensors-22-03906-s001.zip › Supplementary/data/plots/jamming/pdf/pspdr_twanh_box.pdf]

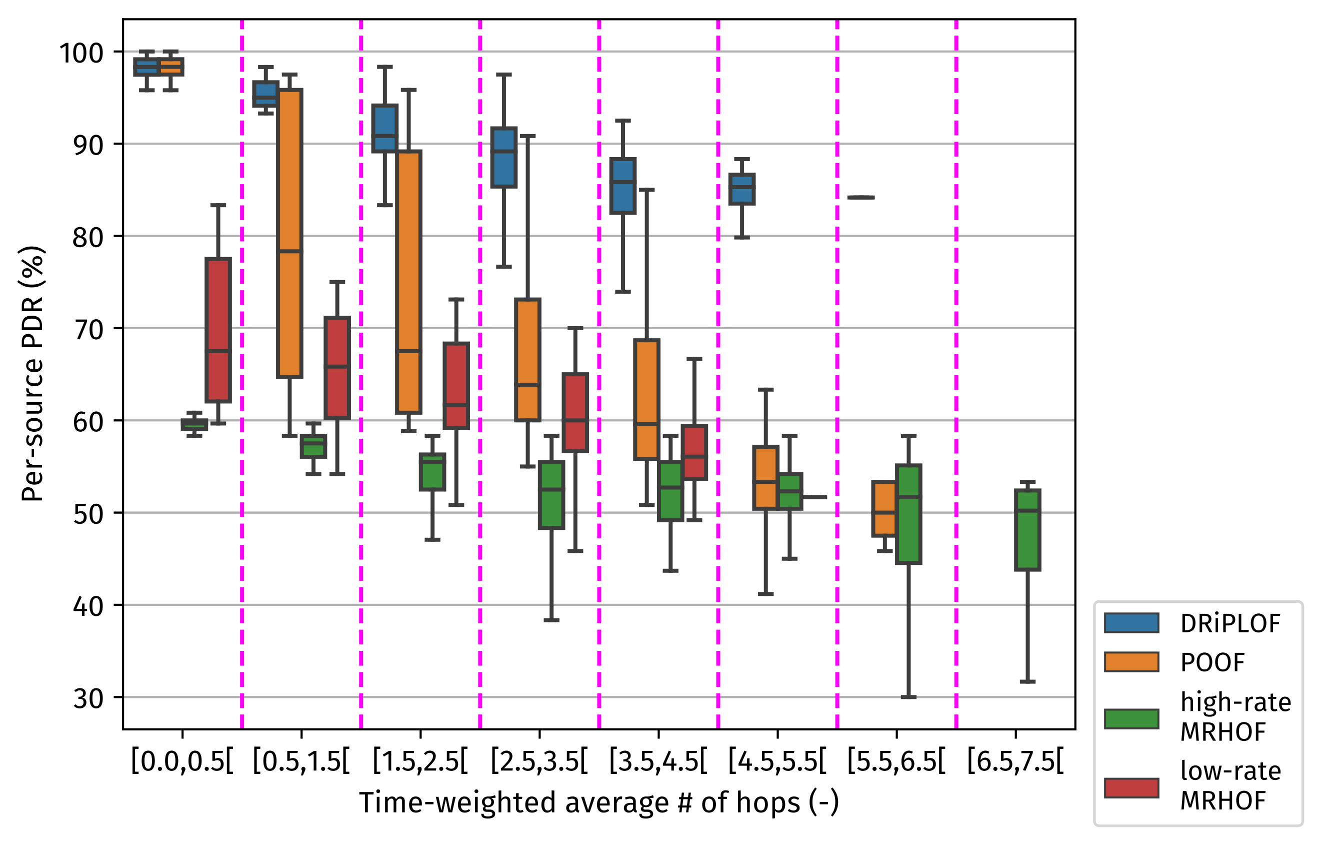

Per-source PDR (%)
100
90
80
70
60
50
40
30
[0.0,0.5[
[0.5,1.5[
[1.5,2.5[
[2.5,3.5[
[3.5,4.5[
[4.5,5.5[
[5.5,6.5[
[6.5,7.5[
Time-weighted average # of hops (-)
DRiPLOF
POOF
high-rate MRHOF
low-rate MRHOF
