# Supplementary material for: DRiPLOF: An RPL Extension for Multi-Interface Wireless Sensor Networks in Interference-Prone Environments
Source: Sensors (Basel). 2022 May 21;22(10):3906. doi: 10.3390/s22103906 (PMC9144939; doi:10.3390/s22103906)
Supplement: Supplementary file 1 [file sensors-22-03906-s001.zip › Supplementary/data/plots/jamming/pdf/twanr_cdf.pdf]

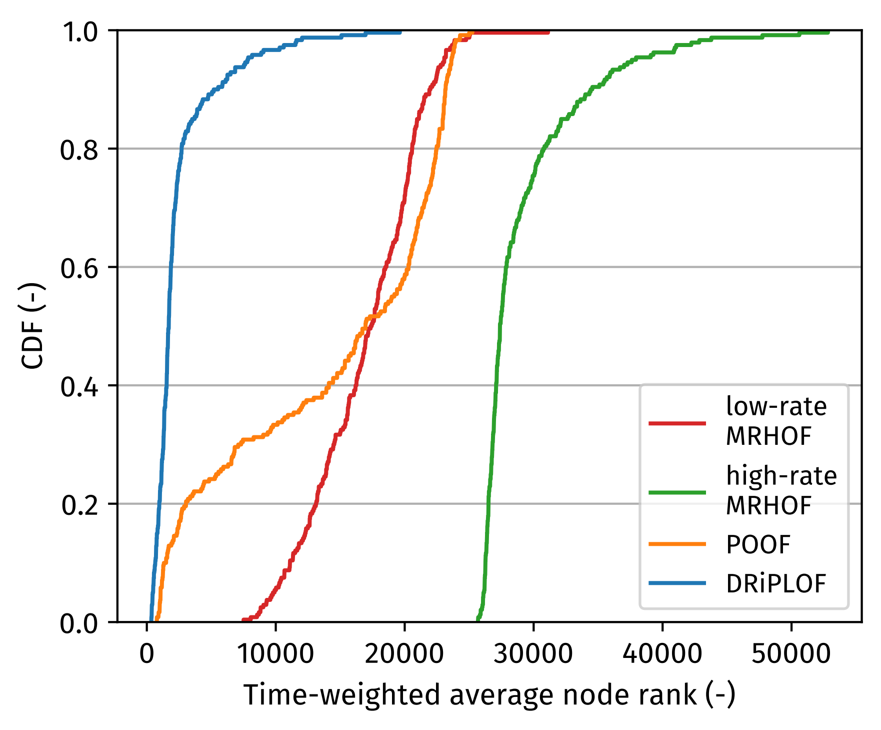

CDF (-)
1.0
0.8
0.6
0.4
0.2
0.0
0
10000
20000
30000
40000
50000
Time-weighted average node rank (-)
low-rate MRHOF
high-rate MRHOF
POOF
DRiPLOF
